# Supplementary material for: Availability of essential, generic medicines before and during COVID-19 at selected public pharmaceutical supply agencies in Ethiopia: a comparative cross-sectional study
Source: BMJ Open. 2024 Mar 4;14(3):e077545. doi: 10.1136/bmjopen-2023-077545 (PMC11146403; doi:10.1136/bmjopen-2023-077545)
Supplement: Author's manuscript [file bmjopen-2023-077545.draft_revisions.pdf]

BMJ Open is committed to open peer review. As part of this commitment we make the peer review history of every article we publish publicly available.

When an article is published we post the peer reviewers' comments and the authors' responses online. We also post the versions of the paper that were used during peer review. These are the versions that the peer review comments apply to.

The versions of the paper that follow are the versions that were submitted during the peer review process. They are not the versions of record or the final published versions. They should not be cited or distributed as the published version of this manuscript.

BMJ Open is an open access journal and the full, final, typeset and author-corrected version of record of the manuscript is available on our site with no access controls, subscription charges or pay-per-view fees (http://bmjopen.bmj.com).

If you have any questions on BMJ Open's open peer review process please email info.bmjopen@bmj.com

BMJ Open

Availability of essential, generic medicines before and during COVID-19 at selected public pharmaceutical supply agencies in Ethiopia: Findings and implications

Journal:	*BMJ Open*
Manuscript ID	bmjopen-2023-077545
Article Type:	Original research
Date Submitted by the Author:	10-Jul-2023
Complete List of Authors:	Melaku , Tsegaye; Jimma University, Institute of Health<br>Mekonnen, Zeleke; Jimma University College of Public Health and Medical Sciences, School of Medical Laboratory Sciences;<br>Terefe Tucho, Gudina; Jimma University<br>Mecha, Mohammed ; Jimma University<br>Årdal, Christine ; Norwegian Institute of Public Health<br>Jahre, Marianne ; BI Norwegian Business School
Keywords:	Health Services Accessibility, HEALTH SERVICES ADMINISTRATION & MANAGEMENT, COVID-19

SCHOLARONE™
Manuscripts

I, the Submitting Author has the right to grant and does grant on behalf of all authors of the Work (as defined in the below author licence), an exclusive licence and/or a non-exclusive licence for contributions from authors who are: i) UK Crown employees; ii) where BMJ has agreed a CC-BY licence shall apply, and/or iii) in accordance with the terms applicable for US Federal Government officers or employees acting as part of their official duties; on a worldwide, perpetual, irrevocable, royalty-free basis to BMJ Publishing Group Ltd ("BMJ") its licensees and where the relevant Journal is co-owned by BMJ to the co-owners of the Journal, to publish the Work in this journal and any other BMJ products and to exploit all rights, as set out in our licence.

The Submitting Author accepts and understands that any supply made under these terms is made by BMJ to the Submitting Author unless you are acting as an employee on behalf of your employer or a postgraduate student of an affiliated institution which is paying any applicable article publishing charge ("APC") for Open Access articles. Where the Submitting Author wishes to make the Work available on an Open Access basis (and intends to pay the relevant APC), the terms of reuse of such Open Access shall be governed by a Creative Commons licence – details of these licences and which Creative Commons licence will apply to this Work are set out in our licence referred to above.

Other than as permitted in any relevant BMJ Author's Self Archiving Policies, I confirm this Work has not been accepted for publication elsewhere, is not being considered for publication elsewhere and does not duplicate material already published. I confirm all authors consent to publication of this Work and authorise the granting of this licence.

For peer review only

Availability of essential, generic medicines before and during COVID-19 at selected public pharmaceutical supply agencies in Ethiopia: Findings and implications

[1]Tsegaye Melaku¥*, [1]Zeleke Mekonnen¥, [1]Gudina Terefe Tucho, [1]Mohammed Mecha, [2]Christine Årdal, [3]Marianne Jahre

[1]Institute of Health, Jimma University, Jimma, Oromia, Ethiopia

[2]Norwegian Institute of Public Health, Oslo, Norway

[3]BI Norwegian Business School, Oslo, Norway

*Corresponding author ¥two authors equally contributed to the work

Address of correspondence

- Email: tsegish.melaku@gmail.com
- Phone: +251913765609

For peer review only

For peer review only - http://bmjopen.bmj.com/site/about/guidelines.xhtml

What is already known on this topic.

- The COVID-19 pandemic has not only affected the global supply of essential medicines but has exacerbated the situation in low-income countries.

What this study adds.

- The impact of COVID-19 pandemic on non-communicable diseases medicines shortages at public pharmaceutical supply agency identified. The stockout situation worsened during this time, indicating challenges in the distribution and supply chain of essential medicines.

How might this study affect research, practice, or policy.

- The study highlights the importance of addressing supply chain disruptions and emphasizes the need for interventions and strategies to strengthen the pharmaceutical supply chain and improve access to these medicines during public health emergencies.

For peer review only - http://bmjopen.bmj.com/site/about/guidelines.xhtml

Abstract

Background: Lockdowns and border closures impacted medicines availability during the COVID-19 pandemic. This study aimed to assess the availability of essential, generic medicines for chronic diseases at selected public pharmaceutical supply agencies in Ethiopia before and during COVID-19. These public agencies supply health facilities, therefore having direct implications on patient access to essential medicines.

Methods: Twenty-two medicines were selected based on the national essential drug list for public health facilities. Seventeen are treatments for cardiovascular disease (CVD) and five for diabetes mellitus (DM). The availability of these products was assessed at two public pharmaceutical supply agency hubs. The study assessed the availability of these medicines on the day of the data collection as well as the records for eight months before the outbreak and one year during the pandemic. Pricing and manufacturers (suppliers) information was also gathered.

Results: Prior to COVID-19, the overall average line-item fill rate for the selected products was 78%, but it dropped to 49% during the pandemic. Ten (10) of the twenty-two (46%) medicines were out of stock on the day of data collection. The drop in the availability of specific medications ranged from 12% to 77% during the pandemic. The average stock out duration for DM and CVD medication was 9 and 11 days per month, respectively before the outbreak. During COVID-19, it was increased to 13 days for DM medications and 17 days for CVD drugs. Although, the prices for some drugs relatively remained stable, there were significant price hikes for some of the products. For example, the unit price of insulin increased by over 130%. The supply distribution did not vary substantially by region during and after COVID-19, with Ethiopian companies supplying over half of the medicines and European suppliers up to 30%.

Conclusion: The COVID-19 pandemic worsened the availability of essential chronic medicines, including higher rates of stockouts and unit price hikes for some products in the study setting. The study's findings imply that the COVID-19 pandemic has aggravated already-existing medicine availability issues. Efforts should be made to develop contingency plans and establish mechanisms to monitor medicine availability and pricing during such crises.

Keywords: Availability, COVID—19, Essential medicines, Manufacturer, Supplier, Stock out

For peer review only - http://bmjopen.bmj.com/site/about/guidelines.xhtml

1. Introduction

Medicines are vital elements to healthcare, and access to medicines is a fundamental human right [1]. The World Health Organization (WHO) defines essential medicines as those which "satisfy the population's priority health care needs" [2]. Drug shortages are commonplace and present significant problems in all countries, including developed nations. The causes of drug shortages are complex and multifaceted, and addressing them requires collaborative effort from various stakeholders. The majority of the developing countries, mostly in sub-Saharan Africa, lack consistent availability and access to essential health products [3, 4]. This is due to various factors including inadequate infrastructure, insufficient funding, currency fluctuations, and more system related factors like poor governance and corruptions [5, 6][7-10].

Although various emergency situations and supply contamination have shut down key manufacturing sites in the past, the COVID-19 pandemic was the first sustained shock event to affect all parts of the global pharmaceutical supply chain [11, 12]. During the pandemic, countries across the world went into lockdowns, shutting down or reducing transport within and between them. This affected the manufacturing, supply, and distribution of medicines, leading to constraints in the medicines supply chain. A general shortage of various types of pharmaceuticals at supply and retail levels has been recorded in many nations, along with price rises and increases in counterfeiting [13, 14]. The impact of the COVID-19 pandemic on the global medical supply chain has been exacerbated by core weaknesses in related to essential medicines production and procurement [15, 16].

Drug shortages caused by the COVID-19 outbreak were mostly fueled by spikes in demand for certain kinds of medications such as medicines used for the management of non-communicable diseases (NCDs) [16-18]. People with NCDs need uninterrupted, reliable access to quality-assured and affordable medicines and health products. During the pandemic service delivery points experienced shortages of medications used to treat cardiovascular disease (CVD) and diabetes mellitus (DM) [18, 19]. Most low-income governments, especially those in sub-Saharan Africa, opt for a distribution

For peer review only - http://bmjopen.bmj.com/site/about/guidelines.xhtml

strategy where they purchase medicines and deliver them to health facilities via a publicly run Central Medical Store (CMS) and a government-owned transport fleet. In recent research, which assessed the availability of the same basket of NCD medicines at the health facility level showed that COVID-19 significantly increased shortages of these products and worsened the stockout situation at the health facilities [20.]. Building on the previous one, this paper describes and analyzes how the COVID-19 pandemic affected supply situations for the same NCD medicines one step prior to health facilities, which is at the pharmaceutical supply agencies.

2. Methods

2.1. Study setting

The study was conducted at two Ethiopian pharmaceutical supply agency (EPSA) hubs (Nekemte and Jimma) in southwestern Ethiopia. Both hubs, Nekemte and Jimma, are approximately 300 kilometers and 350 kilometers from Addis Ababa, respectively. According to the current supply agency clustering scheme, these two hubs are under Western cluster, which is one of the seven clusters consisting of three hubs (Jimma, Nekemte and Gambella). The agency hubs serve as outposts to distribute pharmaceuticals, chemical reagents, medical supplies, and equipment procured by the central office to localities on all corners of the country/region. They also gather pharmaceutical demand within their domain and communicate it with the head office to form an aggregate demand at national level. Hubs have been serving as key governmental structures for the implementation of integrated pharmaceutical and logistic services (IPLS) and distribution of essential health commodities for public health facilities. They also provide supervisory, material support and capacity building to health facilities for strengthening and enforcing the implementation of IPLS and pharmacy services [21] (**diagram 1**). The pharmaceutical supply chain in Ethiopia consists of public and private actors where some of the manufacturing is done in-country. These system mainly comprises of manufacturers/suppliers, wholesalers and/or distributors to the health facilities as the main actors in the pharmaceutical supply chain map (**diagram 2**).

For peer review only - http://bmjopen.bmj.com/site/about/guidelines.xhtml

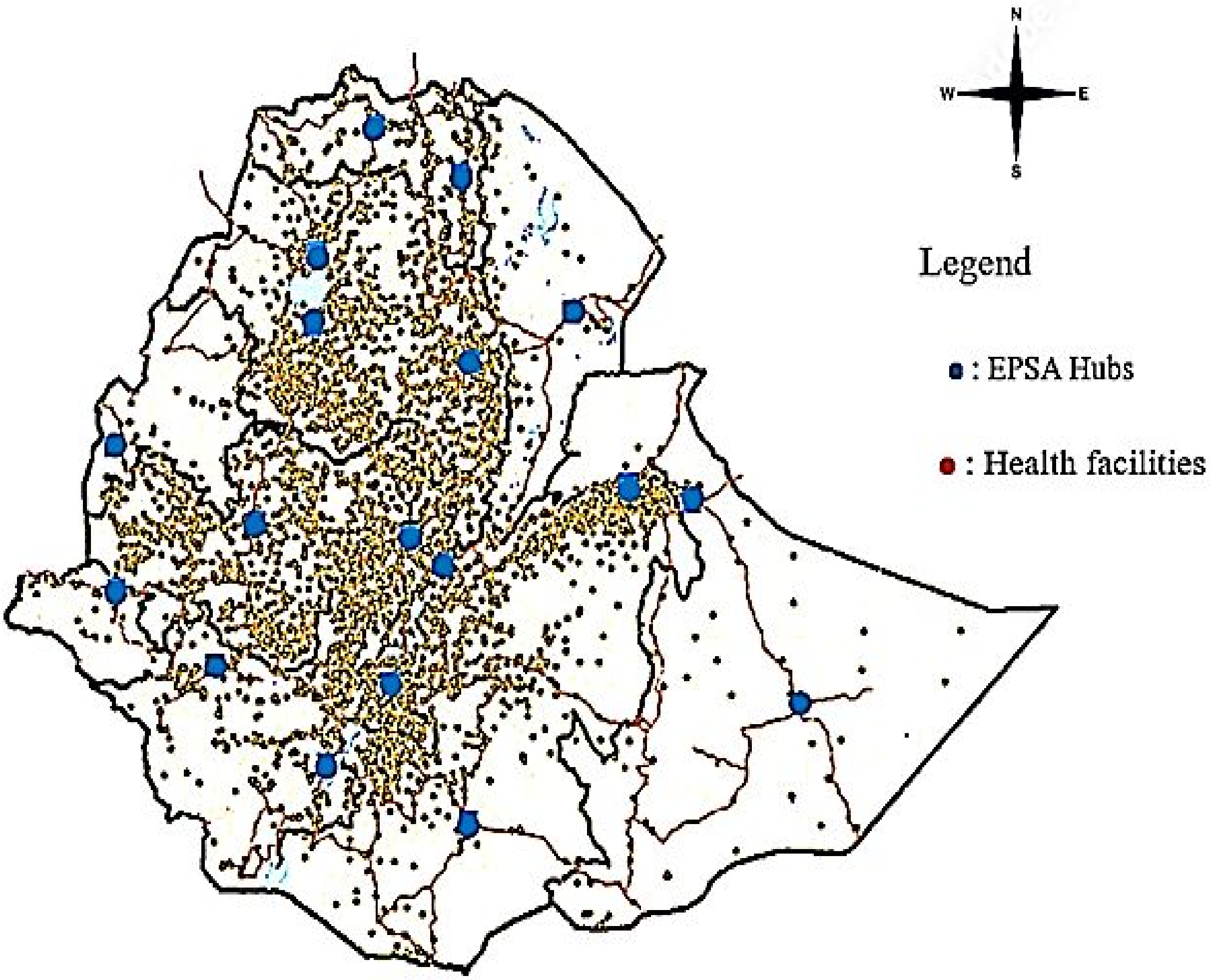

Diagram 1: Map of Ethiopia and location of Ethiopian pharmaceutical supply chain hubs

For peer review only - http://bmjopen.bmj.com/site/about/guidelines.xhtml

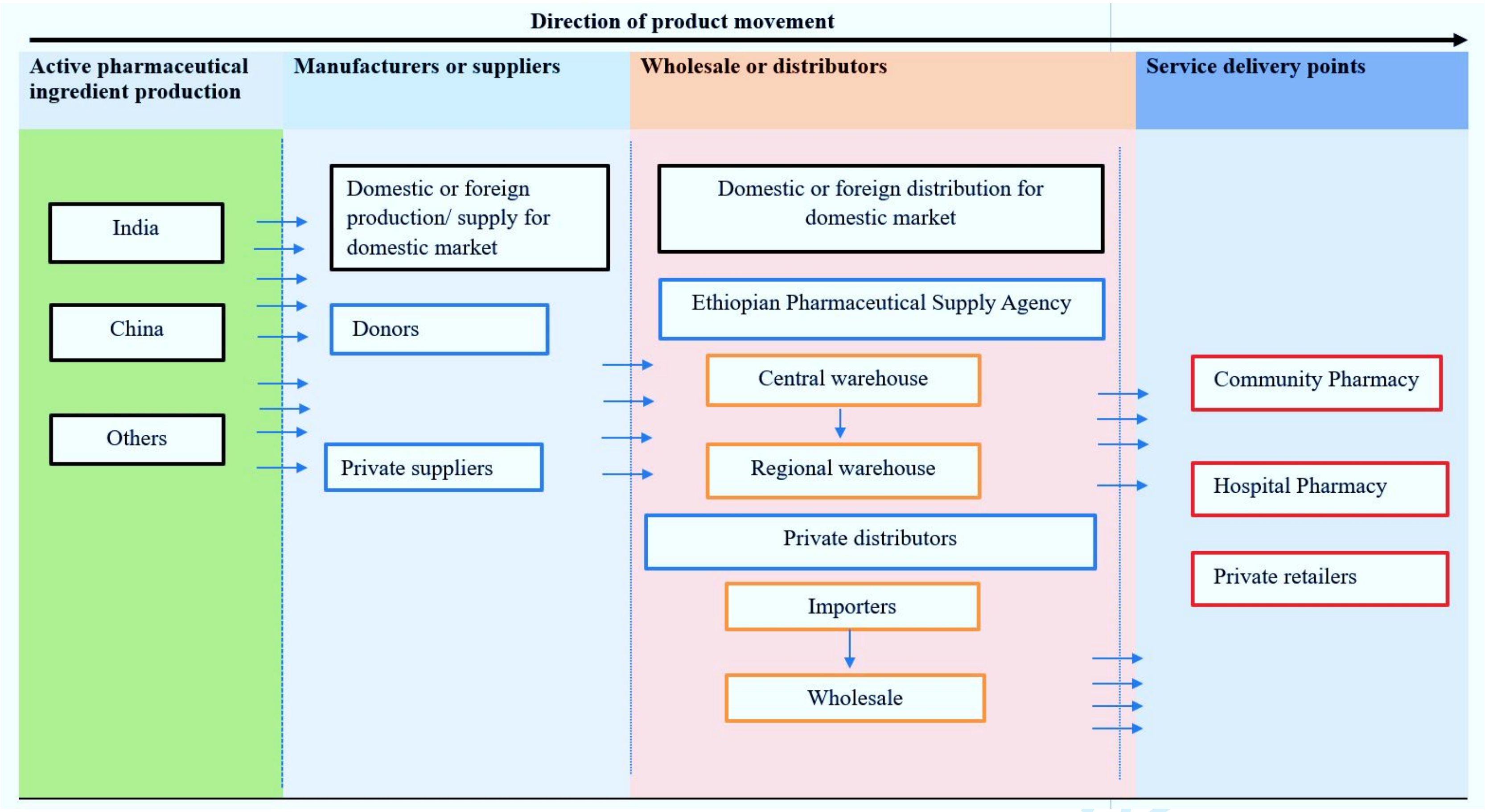

Diagram 2: Pharmaceutical supply chain map of Ethiopian pharmaceutical market

For peer review only - http://bmjopen.bmj.com/site/about/guidelines.xhtml

2.2. Sample selection and time periods

Twenty-two (22) essential, generic medicines were selected from the essential medicines list of the country. Seventeen (17) were cardiovascular disease (CVD) drugs and the remaining five (5) antidiabetics. The products with lowest dosage strength and available formulation of the substance, which have a market authorization, were included. These are the most commonly prescribed medicines and dosages in the health facilities in Ethiopia. The LMIS records were used to track and compare product transactions data for eight months before COVID-19 (between 01 May and 31 December 2019) and for 12 months during pandemic time (between 01 January and 31 December 2020).

2.3. Data collection procedure

Based on the study objective, the modified data collection checklist from the standard tools [22, 23] was used to collect the relevant data. Closed-ended questions about the quantity of medicines ordered, received, distributed, and the stock situation on the day of data collection, were included on the observation checklist. Data was collected through a cross-sectional survey conducted over a period of time during the COVID-19 pandemic. The researchers visited pharmaceutical supply agencies and collected information on the availability of NCD medicines. They also recorded any shortages or stockouts that occurred during this period. A two-day training session was given to the data collectors regarding the study's objectives and how to collect the data. During the data collection process, there was close supervision by the investigators to ensure consistency and completeness of the data. The data was collected from the two public supply agency hubs.

2.4. Data processing and analysis

A descriptive frequency and percentage analysis was conducted to evaluate the line-item fill rate, product availability, and distribution trends before and after the COVID-19 pandemic. The following formulas were used to estimate the current product availability, stock out duration, and line-item fill rate of the selected medicines.

For peer review only - http://bmjopen.bmj.com/site/about/guidelines.xhtml

- Medicine availability = $\frac{\text{Number of product available in a given period}}{\text{The number of product considered}} \times 100$

- Line–item fill rate = the $\frac{\text{amount (quantity) of item received}}{\text{The amount (quantity) of item ordered}} \times 100$

- Average stock–out duration = $\frac{\text{the number of days the product was not available}}{\text{The number of product reviewed}} \times 100$

Based on the WHO's drug availability index, the analysis considered the availability of products that are ordered and received in the supply agencies as well as stock status on the day of data collection. A product is available if it is in stock in the health facility providing the service on the day of the visit or during the specified period. The following ranges were used to describe the medication availability status: less than 30% is very low; 30-49% is low; 50-80% is fairly high; and greater than 80% is very high [24, 25].

For peer review only

For peer review only - http://bmjopen.bmj.com/site/about/guidelines.xhtml

3. Results

3.1. The line-item fill rate

The line-item fill rate indicates how often the EPSA hubs received at least 80% of the quantity ordered for a particular medicine. Prior to COVID-19, the overall average line-item fill rate for the selected products was 78%. It declined to 49% during the pandemic. For antidiabetics, the average line-item fill rate declined from 85% to 56%. For CVD drugs it went down from 76% to 46%. The overall line-fill rate dropped for all selected medicines during COVID-19 (**Figure 1 A and B**).

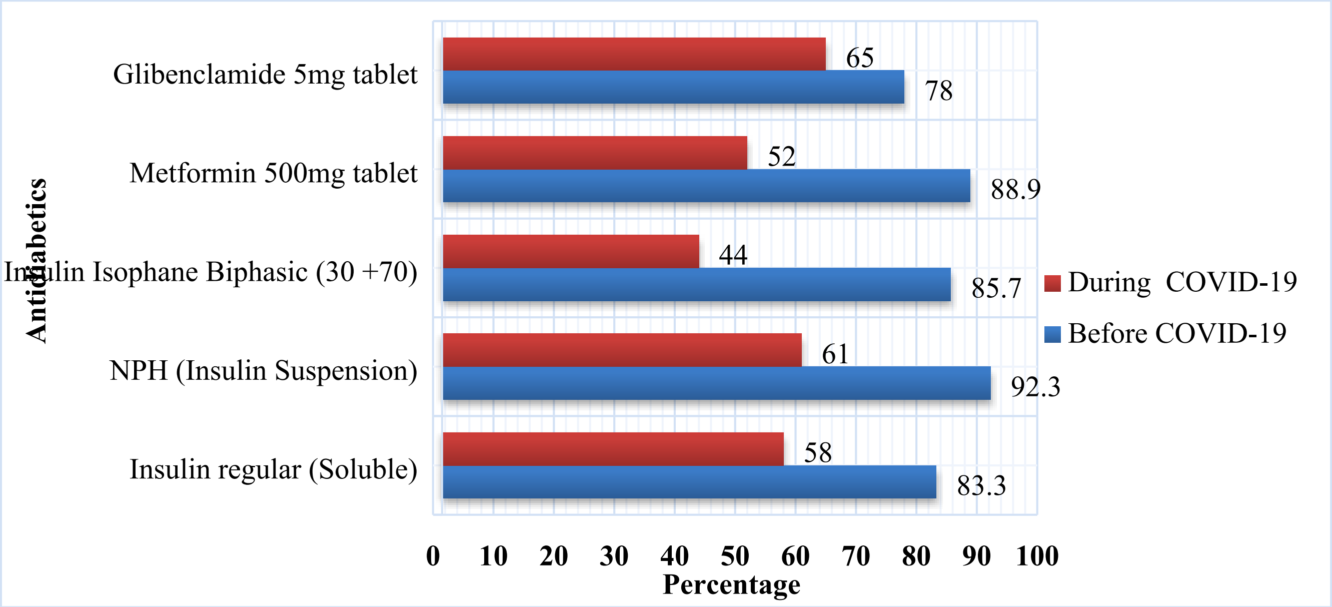

Figure 1A

For peer review only - http://bmjopen.bmj.com/site/about/guidelines.xhtml

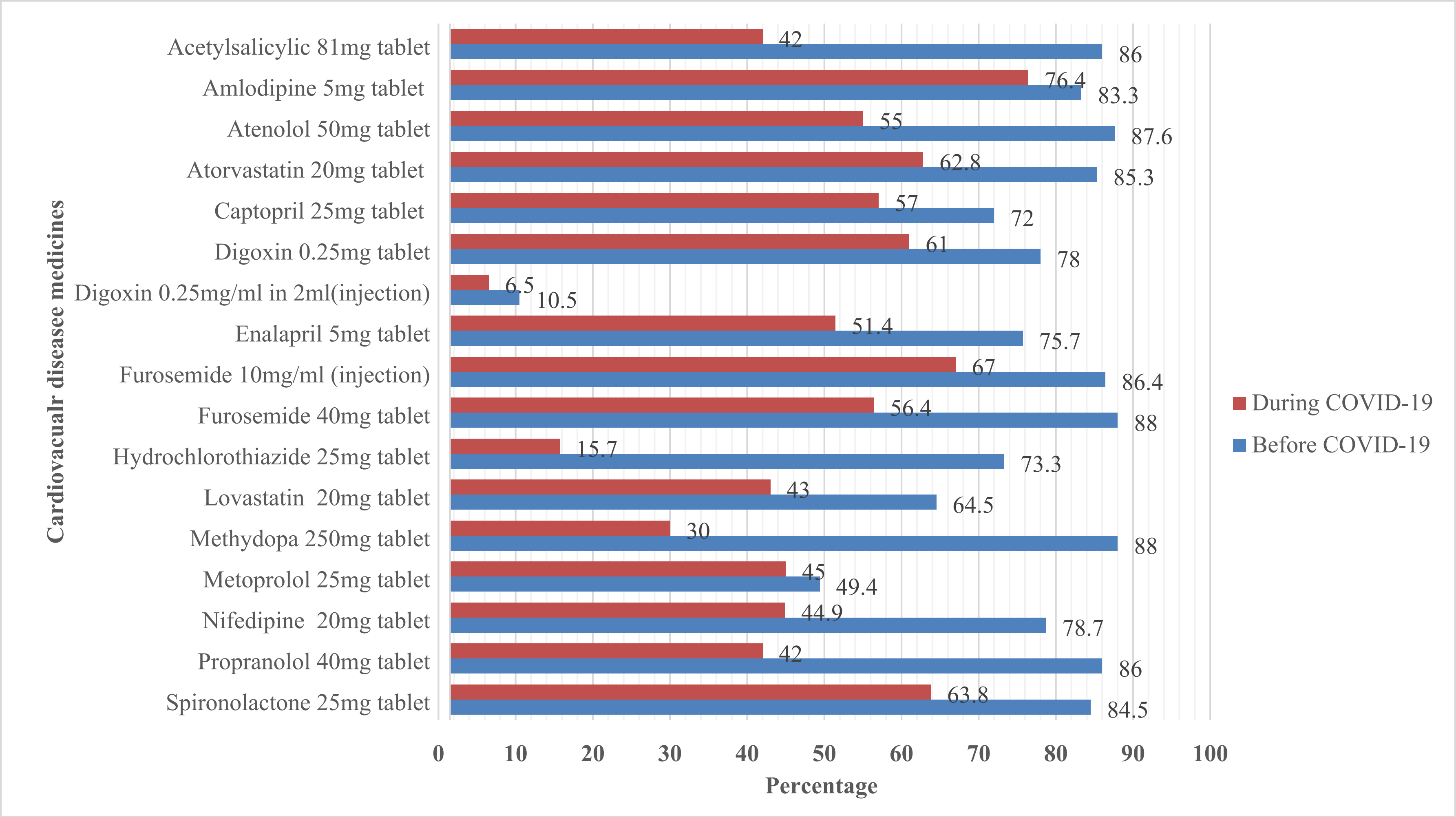

Figure 1B

Figure 1: The average line-item fill rate for selected antidiabetics (Fig 3A) and CVD medications (Fig 3B) before and during COVID-19.

For peer review only - http://bmjopen.bmj.com/site/about/guidelines.xhtml

3.2. Product availability and stock out situation of selected basket medicines

During COVID-19, the overall availability of the selected basket medicines was 49%. On the day of data collection, ten (10) of the twenty-two (46%) were out-of-stock. Before the pandemic, the average stockout duration per month was 9 days for antidiabetics and 11 days for the CVD drugs. This increased to 13 days and 17 days/month, during the pandemic for antidiabetics and the CVD drugs, respectively. The medicines with the least supply were captopril 25mg tablets, metoprolol 25mg tablets, lovastatin 20mg tablets, and digoxin 0.25mg/ml injection. Lovastatin had the longest average stockout of 28 days, closely followed by digoxin injection at 27 days and captopril tablet at 26 days (resulting in WHO availability indices of 11%, 13 % and 23%, respectively). The drop in the availability of specific medications ranged from 12% to 77% during the pandemic. (**Table 1**).

For peer review only - http://bmjopen.bmj.com/site/about/guidelines.xhtml

Table 1: Product availability and average days out of stock for selected antidiabetics and CVD medications before and during COVID-19 pandemic

List of drug products	Pre-pandemic		During the pandemic		Decline in availability (%)
	Average days out of stock	Availability* (%)	Average days out of stock	Availability* (%)	
1. Insulin regular (soluble)	9.5	74.7	13.3	44.6	40
2. NPH insulin (suspension)	8	77.3	10.9	49	37
3. Insulin Isophane (biphasic)	8.5	75.2	12.1	43.4	42
4. Metformin 500 mg tablet	8.7	76.5	12.0	47.3	38
5. Glibenclamide 5mg tablet	10.3	70.6	12.3	56.9	19
6. Aspiring 81mg tablet	9.2	70.8	12.7	44	38
7. Enalapril 5mg tablet	11.0	68	14.4	47	31
8. Captopril 25mg tablet	16.7	56	26	23	59
9. Atenolol 50mg tablet	9.6	73.7	11.8	56.4	24
10. Propranolol 40 mg tablet	9.4	72.6	13.2	44	39
11. Metoprolol 25mg tablet	19	52	21.2	46	12
12. Frusemide 40mg tablet	9.3	71.5	12.4	47.7	33
13. Frusemide 10mg/ml	8.4	76	10	61.5	19
14. Hydrochlorothiazide 25mg tablet	10.8	68	16	35	49
15. Spironolactone 25mg tablet	9.6	74	11	63.5	14
16. Atorvastatin 20mg tablet	15	62	20	41.5	33
17. Lovastatin 20mg tablet	17	45	28	10.5	77
18. Methyldopa 250mg tablet	11.3	66.8	17.2	32	52
19. Amlodipine 5mg tablet	8.7	78	11.2	56	28
20. Nifedipine 20mg tablet	13.5	68.3	19.5	38	44
21. Digoxin 0.25mg tablet	9.6	74	12.4	52.5	29
22. Digoxin 0.25mg/ml in 2ml(injection)	24	31	27	13	58

*based on the WHO availability index [24, 25].

For peer review only - http://bmjopen.bmj.com/site/about/guidelines.xhtml

3.3. Unit price changes of selected antidiabetics and CVD medications

We were unable to assess the unit pricing changes of all medicines because of stockouts for the majority of the products. The prices were relatively stable for some medicines (where there was sufficient data to assess). Most of these products such as metformin glibenclamide, oral furosemide and hydrochlorothiazide were from the local pharmaceutical industries. Yet for some of the products, there were dramatic price increases (**Figure 2**), such as for insulin products and methyldopa. Some of these products imported and supplied by international pharmaceutical supplier such as Novo Nordisk Pharmatech A/S. Likewise, the unit price for oral furosemide, amlodipine and nifedipine increased starting from June 2020. During the study period the value of the Ethiopian birr decreased by 38% compared to USD [26], dramatically increasing the price of imported medicines.

For peer review only

For peer review only - http://bmjopen.bmj.com/site/about/guidelines.xhtml

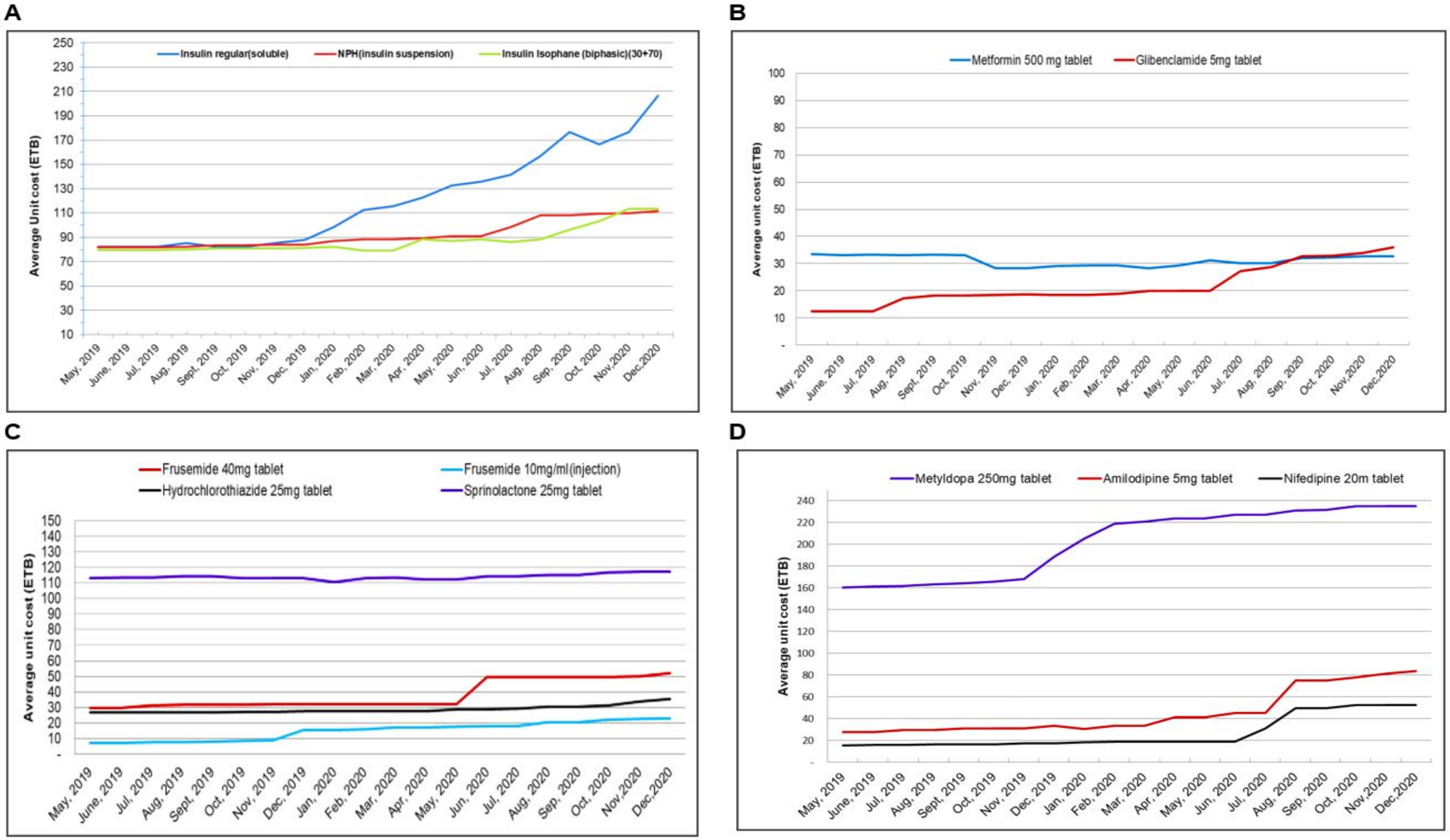

Figure 2: Trends of selected antidiabetics (A, B) and CVD medicines (C, D) unit prices at public pharmaceutical supply agency hubs

For peer review only - http://bmjopen.bmj.com/site/about/guidelines.xhtml

3.4. Selected medicine manufacturer/supplier

This paper uses the terms manufacturers and suppliers interchangeably. Sometimes facilities supply their stock from importer and/or wholesaler, sometimes directly from the manufacturer. For the selected NCD medicines, there were nineteen (19) manufacturers/suppliers over the period. During the eight months pre-pandemic, 192 orders were placed by the two agencies while during the 12 months of the pandemic 264 supply orders were placed. Ethiopian companies represented 50% and 53% of suppliers before and during COVID-19 pandemic, respectively. Similarly, European pharmaceutical industries were supplying 27% prior to COVID-19 and 31% during pandemic, meaning that about 84% NCD medicines at the two agencies were supplied either from Ethiopia or Europe during COVID-19 (**Table 2**).

For peer review only

For peer review only - http://bmjopen.bmj.com/site/about/guidelines.xhtml

Table 2 Lists of medicines manufacturer/supplier during & prior to the pandemic.

S. No	Manufacturers/suppliers name (country)	Percentage of supply orders covered	
		Before COVID-19	During COVID-19
1.	Amino AG (Switzerland)	4	8
2.	Addis Pharmaceutical Factory (Ethiopia)	8	13
3.	AstraZeneca (United Kingdom)	4	4
4.	Bliss GVS Pharma Limited (India)	-	3
5.	Brawn Laboratories Limited (India)	4	1
6.	Cadila Pharmaceuticals PLC (Ethiopia)	10	10
7.	Ciron Drugs & Pharmaceuticals PLC (India)	4	4
8.	East African Pharmaceuticals PLC (Ethiopia)	6	10
9.	EIPICO (Egypt)	4	4
10.	Epharm (Ethiopia)	5	1
11.	Gulf Pharmaceuticals Ltd (UAE)	4	4
12.	Humanwell Pharmaceutical PLC (Ethiopia)	4	1
13.	Intas Pharmaceuticals (India)	2	1
14.	Julphar Pharmaceuticals PLC (Ethiopia)	8	5
15.	Laboratoires Sterop (Belgium)	2	1
16.	Novo Nordisk Pharmatech A/S (Denmark)	4	5
17.	Remedica Ltd (Cyprus)	13	13
18.	Reyoung Pharmaceutical Co., Ltd (China)	4	1
19.	Sansheng Pharmaceutical PLC (Ethiopia)	9	13

Epharm: Ethiopian Pharmaceuticals Manufacturing share company EIPICO: Egyptian International Pharmaceutical Industries Company; UAE: United Arab Emirates

For peer review only

For peer review only - http://bmjopen.bmj.com/site/about/guidelines.xhtml

4. Discussion

Ensuring sustainable availability of essential medicines has been a persistent challenge in Ethiopia. The country has made progress in recent years, but the COVID-19 pandemic highlighted the need for further improvements. More must be done to strengthen the drug supply chain and ensure sustainable availability of essential medicines. This study assessed trends in the availability of selected medicines for chronic diseases at the public supply agencies over a long time-period. We evaluated the fill-item rate, trends, and product availability before and during the pandemic.

The results of the study showed that the COVID-19 pandemic significantly increased shortages of NCD medicines at pharmaceutical supply agencies. The researchers found that the stockout situation worsened during the pandemic, indicating challenges in the distribution and supply chain of essential medicines. In response to the global burden of NCDs, the WHO has developed a Global Action Plan that included target of 80% availability and affordability of essential medicines for the prevention and treatment of DM, CVD, and respiratory diseases [27]. Despite this strong recommendation, the availability of NCD medicines in Ethiopia falls below the recommended target. At national level the median availability of medicines for NCDs was below 50% with average stockout days of up to 27 days [28]. Similarly, the current study showed that the overall availability of selected medicines was 49% during the pandemic, down from 78% pre-pandemic. This includes wide variation, with lovastatin available only 11% according to the WHO availability index during the pandemic. This is consistent with studies from Kenya [29], Portugal [30] , Poland [31] and Congo and Cameroon [32]. This has led to increased difficulties for individuals with chronic conditions to access the medications they need to manage their health. The higher rates of stockouts mean that pharmacies and healthcare facilities are unable to meet the demand for these essential medicines, leaving patients without access to necessary treatments.

Not only were the medicines less available during COVID-19 but some also become more expensive, which is in line with several studies from countries like Bangladesh [41], Portugal [30],Saudi Arabia [42], India [43], Nigeria [44] and Namibia [45]. While the Ethiopian Birr decreased in value compared to the US Dollar by 38%, soluble insulin

For peer review only - http://bmjopen.bmj.com/site/about/guidelines.xhtml

increased by more than 130% in price, which means exchange rates cannot alone explain the price increase. This suggests that other factors, such as supply chain disruptions or increased production costs, may have contributed to the price hike. Moreover, the changes in supplier dynamics on medication availability and affordability might contribute to the price changes during the pandemic. These findings highlight the need for further investigation into the underlying causes of medication price increases during crises. It is crucial to understand the specific factors influencing these price changes to develop effective strategies for mitigating their impact on individuals with chronic conditions. This study also suggests that while there were stable prices for certain medications supplied by local pharmaceutical industries, there were price increases for insulin products and methyldopa, which were imported and supplied by international pharmaceutical suppliers. Factors such as supply chain disruptions, increased demand, and changes in production costs could contribute to these price fluctuations. It is important to further investigate the reasons behind these price increases, as they may have implications for medication affordability and accessibility.

Furthermore, this study also assessed the type of suppliers or manufacturers of selected drugs before and during the epidemic. The supply distribution did not vary substantially by region during and after COVID-19. For NCD medicines it appears that, at least these two public pharmaceutical supply agencies, are mainly supplied by Ethiopian and European companies. The large proportion of Ethiopian suppliers is beneficial, likely both in normal times and in pandemic, as the currency fluctuations should be smaller, although still present due to a likely dependence on imported active pharmaceutical ingredients (APIs). That local pharmaceutical industries were able to step up and meet the demand for these products demonstrates the importance of having strong and resilient local pharmaceutical industries that can provide essential healthcare products during times of crisis. This study highlights the importance of addressing supply chain disruptions during public health emergencies, such as the COVID-19 pandemic, to ensure the availability of essential medicines for patients with NCDs. It emphasizes the need for interventions and strategies to strengthen the pharmaceutical supply chain and improve access to essential medicines in low-income countries. Overall, the COVID-19 pandemic has underscored the critical importance of

For peer review only - http://bmjopen.bmj.com/site/about/guidelines.xhtml

maintaining a robust and resilient healthcare system that can effectively respond to emergencies while ensuring the availability and affordability of essential medicines for those who need them most.

Limitation of the study

To the best of the authors' knowledge, this is one of the first studies assessing supply situations of essential medicines during a pandemic at selected public pharmaceutical supply agencies. Yet, the results may not apply to all medicines. Other therapeutic areas may experience unique trends. The proximity to the capital city may allow for better distribution processes, whereas more remote areas may experience greater challenges. The study included only two facilities, which may have unique characteristics.

5. Conclusion

In summary, significant shortages of selected basket medicines used for the management of DM and CVD were observed at the two selected public pharmaceutical supply agencies. The pandemic has highlighted the importance of ensuring access to essential medications for patients with chronic conditions such as CVD and diabetes. Unit prices of some of the medicines dramatically increased, due to both currency fluctuations and other possible factors. The supply distribution did not vary substantially by region during and after COVID-19, with the two agencies mainly being supplied by Ethiopian and European companies. The findings of this study suggest that the COVID-19 pandemic has exacerbated existing medicines availability problems. Yet it also demonstrates a strong local provision of NCD medicines. Challenges facing these local providers should be further studied to understand how currency fluctuations, dependencies on imported APIs, and other factors influence their ability to supply the local population. It would also be useful to compare these findings with patient experiences to better understand how these stockouts impacted them and their health.

Abbreviations: APIs: Active Pharmaceutical Ingredients; CVD: Cardiovascular Diseases; COVID-19: coronavirus disease 2019; EPSA: Ethiopian Pharmaceutical

For peer review only - http://bmjopen.bmj.com/site/about/guidelines.xhtml

Supply Agency; IRB: Institutional Review Board; SPSS: Statistical Package Software for Social Science; LMICs: low- and middle-income countries; IPLS: Integrated Pharmaceutical and Logistic Services; WHO: World Health Organization, LSAT: Logistics System Assessment Tool; IMAT: Inventory Management Assessment Tool; LIAT: Logistic Indicators Assessment Tool; NCDs: Non-communicable Diseases

Declarations

Ethics approval and consent to participate: Before commencing data collection, ethical approval was obtained from the Institutional Review Board (IRB) of the Institute of Health, Jimma University in Ethiopia (reference number JHRPG/1043/2020). Then, the selected public supply agencies were communicated with a formal letter from the health research and innovation office of Jimma University. The study was conducted after getting permission from the manager of EPSA.

Consent for publication: Not required.

Patient and public involvement: It was not appropriate or possible to involve patients or the public in the design, conduct, or reporting, or dissemination plans of our research

Availability of data and materials: This study included all pertinent data. Additional information could be obtained from the corresponding author upon reasonable request.

Competing interests: None declared.

Funding: This work was supported by the Global health and vaccination research programme

(GLOBVAC), which is the programme of the Research Council of Norway.

Authors' contribution: ZM, TSM, GTT, MM, CÅ and MJ designed the study. ZM and TSM drafted the first version of the manuscript. ZM, TSM, CÅ and MJ discussed the analytic strategy. ZM, TSM and GTT did the data curation and performed the analysis. All authors reviewed the final paper and made creative and substantial contributions, approved the final version, and consented to publication.

For peer review only - http://bmjopen.bmj.com/site/about/guidelines.xhtml

Acknowledgements: We would like to extend our appreciation and thank our data collectors and study participants. We also extend our sincere gratitude for health care professionals, and managers at supply agency hubs for providing us with necessary data/information, for their kind cooperation, and assistance throughout the data collection process. Additionally, we would like to express our heartfelt gratitude to the Global health and vaccination research programme (GLOBVAC) for financially supporting this research within the auspices of the MIA (Measures for Improved Availability of Medicines and Vaccines (The MIA Project | BI). Finally, our acknowledgment extends to the Institute of health of Jimma University for supporting us in the ethical approval process and in the official communication of respective office and health facilities.

References

1. World Health Organization (WHO, 2006), Hogerzeil, H.V., *Essential medicines and human rights: what can they learn from each other?* Bulletin of the World Health Organization, 2006. **84**: p. 371-375.
2. De Weerdt, E., et al., *Toward a European definition for a drug shortage: a qualitative study.* Frontiers in pharmacology, 2015. **6**: p. 253.
3. Wagenaar, B.H., et al., *Stock‐outs of essential health products in M ozambique–longitudinal analyses from 2011 to 2013.* Tropical Medicine & International Health, 2014. **19**(7): p. 791-801.
4. Wild, L. and D. Cammack, *The supply and distribution of essential medicines in Malawi.* 2013.
5. Kohler, J.C., et al., *Corruption in the pharmaceutical sector: diagnosing the challenges.* 2016.
6. Martinez, M.G., J.C. Kohler, and H. McAlister, *Corruption in the pharmaceutical sector*, in *The Handbook of Business and Corruption*. 2017, Emerald Publishing Limited. p. 329-361.
7. Besancon, L. and B. Chaar, *Report of the International Summit on medicines shortage.* Toronto, Canada: International Pharmaceutical Federation, 2013.
8. Morrison, A., *Drug supply disruptions [environmental scan issue 17].* Ottawa: Canadian Agency for Drugs and Technologies in Health. Available online at: https://www. cadth. ca/drug-supply-disruptions, 2011.
9. World Health Organization (WHO, 2017)., *Access to medicines: making market forces serve the poor.* Geneva, Switzerland, 2017.
10. Ahlqvist, V., et al., *Supply chain risk management strategies in normal and abnormal times: policymakers' role in reducing generic medicine shortages.* International Journal of Physical Distribution & Logistics Management, 2023. **53**(2): p. 206-230.

For peer review only - http://bmjopen.bmj.com/site/about/guidelines.xhtml

11. Magableh, G.M., *Supply chains and the COVID-19 pandemic: A comprehensive framework.* European Management Review, 2021. **18**(3): p. 363-382.
12. Yu, Z., et al., *Disruption in global supply chain and socio-economic shocks: a lesson from COVID-19 for sustainable production and consumption.* Operations Management Research, 2021: p. 1-16.
13. Ziavrou, K.S., S. Noguera, and V.A. Boumba, *Trends in counterfeit drugs and pharmaceuticals before and during COVID-19 pandemic.* Forensic Science International, 2022: p. 111382.
14. Noguera, S. and K. Ziavrou, *With the world in turmoil, transnational organized crime sees opportunity in counterfeit pharmaceuticals and medical supplies during the COVID-19 pandemic.* Perspectives, 2020. **12**.
15. Ozili, P.K. and T. Arun, *Spillover of COVID-19: impact on the Global Economy.* Available at SSRN 3562570, 2020.
16. Bookwalter, C.M., *Drug shortages amid the COVID-19 pandemic.* Us Pharm, 2021. **46**(2): p. 25-8.
17. O World Health Organization (WHO, 2023),, *COVID-19 pandemic significantly impacted access to medicines for noncommunicable diseases; https://www.who.int/news/item/22-03-2023-covid-19-pandemic-significantly-impacted-access-to-medicines-for-noncommunicable-diseases; access date 20 April 2023.* March 2023.
18. World Health Organization (WHO, 2023), *Access to NCD medicines: emergent issues during the COVID-19 pandemic and key structural factors.* 2023.
19. Sen-Crowe, B., M. McKenney, and A. Elkbuli, *Medication shortages during the COVID-19 pandemic: Saving more than COVID lives.* The American journal of emergency medicine, 2021. **45**: p. 557.
20. Mekonnen, Z., et al., The knock-on effects of COVID-19 pandemic on the supply and availability of generic medicines in Ethiopia: mixed methods study. BMC Health Services Research, 2023. 23(1): p. 1-16.
21. Agency, E.P.S., *Info Hubs - Ethiopian Pharmaceuticals Supply Agency(EPSA); https://epss.gov.et/info-hubs/; Accessed date: May, 22, 2022.* 2021.
22. USAID and T.O. DELIVER PROJECT, *Logistics Indicators Assessment Tool (LIAT)*. 2008, USAID Arlington, VA.
23. USAID and T.O. DELIVER PROJECT, *Logistics System Assessment Tool (LSAT)*. 2009, USAID| DELIVER PROJECT, Task Order 1, Arlington.
24. World Health Organization (WHO, 2008),, *Measuring medicine prices, availability, affordability and price components*. 2008, World Health Organization.
25. World Health Organization (WHO, 2017), *Technical definitions of shortages and Stockouts of medicines and vaccines.* Geneva: World Health Organization, 2017.
26. Exchange-Rates.org, *US Dollar (USD) To Ethiopian Birr (ETB) Exchange Rate History for December 31, 2019; https://www.exchange-rates.org/exchange-rate-history/usd-etb-2019-12-31. accessed date: 20 May 2023.* 2023.
27. World Health Organization (WHO, 2013), *Global action plan for the prevention and control of noncommunicable diseases 2013-2020*. 2013: World Health Organization.
28. ETHiopian Ministry of Health, FDRE, 2003 *Assessment of the pharmaceutical sector in Ethiopia*. 2003, Addis Ababa, Ethiopia.: Ministry of Health.

For peer review only - http://bmjopen.bmj.com/site/about/guidelines.xhtml

29. Kiragu, Z.W., et al., *Household access to non-communicable disease medicines during universal health care roll-out in Kenya: A time series analysis.* PloS one, 2022. **17**(4): p. e0266715.
30. Romano, S., et al., *Time-trend analysis of medicine sales and shortages during COVID-19 outbreak: Data from community pharmacies.* Research in Social and Administrative Pharmacy, 2021. **17**(1): p. 1876-1881.
31. Nowak, B.M., et al., *Availability of Cardiodiabetological Drugs in Poland during the First Year of COVID-19 Pandemic: Retrospective Study.* BioMed, 2022. **2**(1): p. 117-126.
32. Schäfermann, S., et al., *Availability, prices and affordability of selected antibiotics and medicines against non-communicable diseases in western Cameroon and northeast DR Congo.* Plos one, 2020. **15**(1): p. e0227515.
33. Attaei, M.W., et al., *Availability and affordability of blood pressure-lowering medicines and the effect on blood pressure control in high-income, middle-income, and low-income countries: an analysis of the PURE study data.* The Lancet Public Health, 2017. **2**(9): p. e411-e419.
34. Ewen, M., et al., *Baseline assessment of WHO's target for both availability and affordability of essential medicines to treat non-communicable diseases.* PloS one, 2017. **12**(2): p. e0171284.
35. Cameron, A., et al., *Medicine prices, availability, and affordability in 36 developing and middle-income countries: a secondary analysis.* The lancet, 2009. **373**(9659): p. 240-249.
36. Mendis, S., et al., *The availability and affordability of selected essential medicines for chronic diseases in six low-and middle-income countries.* Bulletin of the world health organization, 2007. **85**(4): p. 279-288.
37. van Mourik, M.S., et al., *Availability, price and affordability of cardiovascular medicines: a comparison across 36 countries using WHO/HAI data.* BMC Cardiovascular disorders, 2010. **10**(1): p. 1-9.
38. Husain, M.J., et al., *Access to cardiovascular disease and hypertension medicines in developing countries: an analysis of essential medicine lists, price, availability, and affordability.* Journal of the American Heart Association, 2020. **9**(9): p. e015302.
39. Das, M., *Lebanon faces critical shortage of drugs.* The Lancet Oncology, 2021. **22**(8): p. 1063.
40. (ADA), A.D.A., *NEW DATA ALERT: COVID-19 Brings Crisis of Access for Millions Living with Diabetes;* https://diabetes.org/newsroom/press-releases/COVID-19-brings-crisis-of-access-for-millions-living-with-diabetes *Acceessed date 15 January 2023.* December 23, 2020.
41. Haque, M., et al., *Availability and price changes of potential medicines and equipment for the prevention and treatment of COVID-19 among pharmacy and drug stores in Bangladesh; findings and implications.* Bangladesh Journal of Medical Science, 2020: p. 36-S 50.
42. Aljadeed, R., et al. *The impact of COVID-19 on essential medicines and personal protective equipment availability and prices in Saudi Arabia*. in *Healthcare*. 2021. MDPI.

For peer review only - http://bmjopen.bmj.com/site/about/guidelines.xhtml

43. Haque, M., et al., *Utilisation, availability and price changes of medicines and protection equipment for COVID-19 among selected regions in India: findings and implications.* Frontiers in pharmacology, 2021. **11**: p. 582154.
44. Awucha, N.E., et al., *Impact of the COVID-19 pandemic on consumers' access to essential medicines in Nigeria.* The American journal of tropical medicine and hygiene, 2020. **103**(4): p. 1630.
45. Tirivangani, T., et al., *Impact of COVID-19 pandemic on pharmaceutical systems and supply chain–a phenomenological study.* Exploratory Research in clinical and social pharmacy, 2021. **2**: p. 100037.
46. Khader, M.A., T. Jabeen, and R. Namoju, *A cross sectional study reveals severe disruption in glycemic control in people with diabetes during and after lockdown in India.* Diabetes & Metabolic Syndrome: Clinical Research & Reviews, 2020. **14**(6): p. 1579-1584.
47. Singh, K., et al., *Health, psychosocial, and economic impacts of the COVID-19 pandemic on people with chronic conditions in India: a mixed methods study.* BMC public health, 2021. **21**: p. 1-15.
48. De Vet, J.M., et al., *Impacts of the COVID-19 pandemic on EU industries*. 2021: European Parliament Strasbourg, France.
49. Bucher, A. and G.B. Wolff, *Increasing the global supply of essential medical supplies: Time for Europe to step up its global leadership.* Bruegel-Blogs, 2021: p. NA-NA.
50. Gereffi, G., *What does the COVID-19 pandemic teach us about global value chains? The case of medical supplies.* Journal of International Business Policy, 2020. **3**: p. 287-301.
51. Zamiela, C., N.U.I. Hossain, and R. Jaradat, *Enablers of resilience in the healthcare supply chain: A case study of US healthcare industry during COVID-19 pandemic.* Research in Transportation Economics, 2022. **93**: p. 101174.
52. Express, F., *J.U. Haroon, Pharma Industry Growth Halves in 2020; https://www.thefinancialexpress.com.bd/trade/pharma-industry-growth-halves-in-2020-1610159516 Accessed date 03 January, 2023.* 2020.
53. Elsafty, A. and M. Osman, *The Impact of COVID-19 on the Efficiency of Packing Lines in Pharmaceutical Manufacturing Sites in Egypt.* International Journal of Business and Management, 2021. **16**(7): p. 57-72.
54. Joshi, A., P. Bhaskar, and P.K. Gupta, *Indian economy amid COVID-19 lockdown: A prespective.* J. Pure Appl. Microbiol, 2020. **14**: p. 957-961.
55. Purwanto, A., *The Covid-19 pandemic impact on industries performance: an explorative study of Indonesian companies.* Journal of Critical Reviews, 2020.
56. Abdelhadi, A. and E. Akkartal, *The Impact Of COVID-19 on Global Supply Chain.*
57. Lozano-Diez, J., J. Marmolejo-Saucedo, and R. Rodriguez-Aguilar, *Designing a resilient supply chain: An approach to reduce drug shortages in epidemic outbreaks.* EAI Endorsed Transactions on Pervasive Health and Technology, 2020. **6**(21): p. e2.
58. Adebisi, Y., A. Jumoke, and O. Carolyn, *Coronavirus disease-19 and access to medicines in Africa.* International Journal of Health & Allied Sciences, 2020(5): p. 120-120.

For peer review only - http://bmjopen.bmj.com/site/about/guidelines.xhtml

59. Wirtz, V.J., et al., *Essential medicines for universal health coverage.* The Lancet, 2017. **389**(10067): p. 403-476.
60. Suleman, S., et al., *Pharmaceutical regulatory framework in Ethiopia: a critical evaluation of its legal basis and implementation.* Ethiopian journal of health sciences, 2016. **26**(3): p. 259-276.
61. Ewen, M., et al., *Prices and availability of locally produced and imported medicines in Ethiopia and Tanzania.* Journal of pharmaceutical policy and practice, 2017. **10**(1): p. 1-9.
62. Marew, T., et al., *Trends and Challenges in Access to Essential Medicines in Ethiopia and the Contributions of Local Pharmaceutical Production.* Ethiopian Journal of Health Sciences, 2022. **32**(5): p. 1027-1042.

For peer review only

For peer review only - http://bmjopen.bmj.com/site/about/guidelines.xhtml

STROBE Statement—Checklist of items that should be included in reports of ***cross-sectional studies***

	Item No	Recommendation	Page No
**Title and abstract**	1	(*a*) Indicate the study's design with a commonly used term in the title or the abstract	1
		(*b*) Provide in the abstract an informative and balanced summary of what was done and what was found	3
**Introduction**			
Background/rationale	2	Explain the scientific background and rationale for the investigation being reported	4
Objectives	3	State specific objectives, including any prespecified hypotheses	5
**Methods**			
Study design	4	Present key elements of study design early in the paper	5
Setting	5	Describe the setting, locations, and relevant dates, including periods of recruitment, exposure, follow-up, and data collection	5
Participants	6	(*a*) Give the eligibility criteria, and the sources and methods of selection of participants	5-6
Variables	7	Clearly define all outcomes, exposures, predictors, potential confounders, and effect modifiers. Give diagnostic criteria, if applicable	5-7
Data sources/ measurement	8*	For each variable of interest, give sources of data and details of methods of assessment (measurement). Describe comparability of assessment methods if there is more than one group	*8*
Bias	9	Describe any efforts to address potential sources of bias	NA
Study size	10	Explain how the study size was arrived at	8
Quantitative variables	11	Explain how quantitative variables were handled in the analyses. If applicable, describe which groupings were chosen and why	8-9
Statistical methods	12	(*a*) Describe all statistical methods, including those used to control for confounding	8-9
		(*b*) Describe any methods used to examine subgroups and interactions	8-9
		(*c*) Explain how missing data were addressed	
		(*d*) If applicable, describe analytical methods taking account of sampling strategy	NA
		(*e*) Describe any sensitivity analyses	NA
**Results**			
Participants	13*	(a) Report numbers of individuals at each stage of study—eg numbers potentially eligible, examined for eligibility, confirmed eligible, included in the study, completing follow-up, and analysed	10
		(b) Give reasons for non-participation at each stage	10
		(c) Consider use of a flow diagram	NA
Descriptive data	14*	(a) Give characteristics of study participants (eg demographic, clinical, social) and information on exposures and potential confounders	10
		(b) Indicate number of participants with missing data for each variable of interest	NA
Outcome data	15*	Report numbers of outcome events or summary measures	
Main results	16	(*a*) Give unadjusted estimates and, if applicable, confounder-adjusted estimates and their precision (eg, 95% confidence interval). Make clear which confounders were adjusted for and why they were included	10-17

For peer review only

		(*b*) Report category boundaries when continuous variables were categorized	NA
		(*c*) If relevant, consider translating estimates of relative risk into absolute risk for a meaningful time period	NA
Other analyses	17	Report other analyses done—eg analyses of subgroups and interactions, and sensitivity analyses	NA
**Discussion**			
Key results	18	Summarise key results with reference to study objectives	18-20
Limitations	19	Discuss limitations of the study, taking into account sources of potential bias or imprecision. Discuss both direction and magnitude of any potential bias	20
Interpretation	20	Give a cautious overall interpretation of results considering objectives, limitations, multiplicity of analyses, results from similar studies, and other relevant evidence	20
Generalisability	21	Discuss the generalisability (external validity) of the study results	20
**Other information**			
Funding	22	Give the source of funding and the role of the funders for the present study and, if applicable, for the original study on which the present article is based	21

*Give information separately for exposed and unexposed groups.

Note: An Explanation and Elaboration article discusses each checklist item and gives methodological background and published examples of transparent reporting. The STROBE checklist is best used in conjunction with this article (freely available on the Web sites of PLoS Medicine at http://www.plosmedicine.org/, Annals of Internal Medicine at http://www.annals.org/, and Epidemiology at http://www.epidem.com/). Information on the STROBE Initiative is available at www.strobe-statement.org.

For peer review only

For peer review only - http://bmjopen.bmj.com/site/about/guidelines.xhtml

BMJ Open

Availability of essential, generic medicines before and during COVID-19 at selected public pharmaceutical supply agencies in Ethiopia: Comparative cross-sectional study

Journal:	*BMJ Open*
Manuscript ID	bmjopen-2023-077545.R1
Article Type:	Original research
Date Submitted by the Author:	14-Dec-2023
Complete List of Authors:	Melaku , Tsegaye; Jimma University, Institute of Health<br>Mekonnen, Zeleke; Jimma University College of Public Health and Medical Sciences, School of Medical Laboratory Sciences;<br>Terefe Tucho, Gudina; Jimma University<br>Mecha, Mohammed ; Jimma University<br>Årdal, Christine ; Norwegian Institute of Public Health<br>Jahre, Marianne ; BI Norwegian Business School
**Primary Subject Heading**:	Public health
Secondary Subject Heading:	Cardiovascular medicine, Global health, Health services research, Pharmacology and therapeutics, Patient-centred medicine
Keywords:	Health Services Accessibility, HEALTH SERVICES ADMINISTRATION & MANAGEMENT, COVID-19

SCHOLARONE™
Manuscripts

I, the Submitting Author has the right to grant and does grant on behalf of all authors of the Work (as defined in the below author licence), an exclusive licence and/or a non-exclusive licence for contributions from authors who are: i) UK Crown employees; ii) where BMJ has agreed a CC-BY licence shall apply, and/or iii) in accordance with the terms applicable for US Federal Government officers or employees acting as part of their official duties; on a worldwide, perpetual, irrevocable, royalty-free basis to BMJ Publishing Group Ltd ("BMJ") its licensees and where the relevant Journal is co-owned by BMJ to the co-owners of the Journal, to publish the Work in this journal and any other BMJ products and to exploit all rights, as set out in our licence.

The Submitting Author accepts and understands that any supply made under these terms is made by BMJ to the Submitting Author unless you are acting as an employee on behalf of your employer or a postgraduate student of an affiliated institution which is paying any applicable article publishing charge ("APC") for Open Access articles. Where the Submitting Author wishes to make the Work available on an Open Access basis (and intends to pay the relevant APC), the terms of reuse of such Open Access shall be governed by a Creative Commons licence – details of these licences and which Creative Commons licence will apply to this Work are set out in our licence referred to above.

Other than as permitted in any relevant BMJ Author's Self Archiving Policies, I confirm this Work has not been accepted for publication elsewhere, is not being considered for publication elsewhere and does not duplicate material already published. I confirm all authors consent to publication of this Work and authorise the granting of this licence.

For peer review only

Availability of essential, generic medicines before and during COVID-19 at selected public pharmaceutical supply agencies in Ethiopia: Comparative cross-sectional study

[1]Tsegaye Melaku¥*, [1]Zeleke Mekonnen¥, [1]Gudina Terefe Tucho, [1]Mohammed Mecha, [2]Christine Årdal, [3]Marianne Jahre

[1]Institute of Health, Jimma University, Jimma, Oromia, Ethiopia
[2]Norwegian Institute of Public Health, Oslo, Norway
[3]BI Norwegian Business School, Oslo, Norway

*Corresponding author ¥two authors equally contributed to the work

Address of correspondence

- Email: tsegish.melaku@gmail.com
- Phone: +251913765609

For peer review only - http://bmjopen.bmj.com/site/about/guidelines.xhtml

Abstract

Objectives Lockdowns and border closures impacted medicines availability during the COVID-19 pandemic. This study aimed to assess the availability of essential, generic medicines for chronic diseases at public pharmaceutical supply agencies in Ethiopia.

Design Comparative cross-sectional study

Setting The availability of essential, generic medicines for chronic diseases was assessed at two public pharmaceutical supply agency hubs.

Participants The current study included public supply agency hub managers, warehouse managers and forecasting officers at the study setting.

Outcomes The assessment encompassed the availability of chronic medicines on the day of data collection, as well as records spanning eight months before the outbreak and one year during the pandemic. A total of twenty-two medicines were selected based on their inclusion in the national essential drug list for public health facilities, including seventeen medicines for cardiovascular disease and five for diabetes mellitus.

Results The results of the study indicate that the mean availability of the selected basket medicines was 43.3% (95% CI: 37.1-49.5) during COVID-19, which was significantly lower than the availability of 67.4% (95% CI: 62.2-72.6) before the outbreak (P<0.001). Prior to COVID-19, the overall average line-item fill rate for the selected products was 78%, but it dropped to 49% during the pandemic. Furthermore, the mean number of days out of stock was 11.7 (95% CI: 9.9-13.5) before the outbreak of COVID-19, which significantly increased to 15.7 (95% CI: 13.2-18.2) during the pandemic, indicating a statistically significant difference (P<0.001).

Conclusion The COVID-19 pandemic worsened the availability of essential chronic medicines, including higher rates of stockouts and unit price hikes for some products in the study setting. The study's findings imply that the COVID-19 pandemic has aggravated already-existing medicine availability issues. Efforts should be made to develop contingency plans and establish mechanisms to monitor medicine availability and pricing during such crises.

Keywords: Availability, COVID—19, Essential medicines, Manufacturer, Supplier, Stock out

For peer review only - http://bmjopen.bmj.com/site/about/guidelines.xhtml

Strengths and limitations of this study

- It provides a comprehensive assessment of the availability of essential, generic medicines before and during COVID-19.
- It uses a standardized methodology for data collection, ensuring consistency and reliability of results.
- It is limited to a specific geographic location and may not be representative of the entire country.

1. Introduction

Medicines are vital elements to healthcare, and access to medicines is a fundamental human right [1]. The World Health Organization (WHO) defines essential medicines as those which "satisfy the population's priority health care needs" [2]. Drug shortages are commonplace and present significant problems in all countries, including developed nations. The causes of drug shortages are complex and multifaceted, and addressing them requires collaborative effort from various stakeholders. The majority of the developing countries, mostly in sub-Saharan Africa, lack consistent availability and access to essential health products [3, 4]. This is due to various factors including inadequate infrastructure, insufficient funding, currency fluctuations, and more system related factors like poor governance and corruptions [5, 6][7-10].

Although various emergency situations and supply contamination have shut down key manufacturing sites in the past, the COVID-19 pandemic was the first sustained shock event to affect all parts of the global pharmaceutical supply chain [11, 12]. During the pandemic, countries across the world went into lockdowns, shutting down or reducing transport within and between them. This affected the manufacturing, supply, and distribution of medicines, leading to constraints in the medicines supply chain. A general shortage of various types of pharmaceuticals at supply and retail levels has been recorded in many nations, along with price rises and increases in falsified products [13, 14]. The impact of the COVID-19 pandemic on the global medical supply chain has been exacerbated by core weaknesses in relation to essential medicines production and procurement [15, 16].

For peer review only - http://bmjopen.bmj.com/site/about/guidelines.xhtml

Drug shortages caused by the COVID-19 outbreak were mostly fueled by spikes in demand for certain kinds of medications such as medicines used for the management of non-communicable diseases (NCDs) [16-18]. People with NCDs need uninterrupted, reliable access to quality-assured and affordable medicines and health products. During the pandemic service delivery points experienced shortages of medications used to treat cardiovascular disease (CVD) and diabetes mellitus (DM) [18, 19]. Most low-income governments, especially those in sub-Saharan Africa, opt for a distribution strategy where they purchase medicines and deliver them to health facilities via a publicly run Central Medical Store (CMS) and a government-owned transport fleet. In recent research, which assessed the availability of the same basket of NCD medicines at the health facility level showed that COVID-19 significantly increased shortages of these products and worsened the stockout situation at the health facilities [20.]. Expanding upon its antecedent, the present manuscript delineates and scrutinizes the impact of the COVID-19 pandemic on the supply chain dynamics of non-communicable disease (NCD) medications at the level of pharmaceutical supply agencies, immediately preceding their distribution to healthcare facilities.

2. Methods

2.1. Study setting

The study was conducted at two Ethiopian pharmaceutical supply agency (EPSA) hubs (Nekemte and Jimma) in southwestern Ethiopia. Both hubs, Nekemte and Jimma, are approximately 300 kilometers and 350 kilometers from Addis Ababa, respectively and about 200 kilometers from each other. According to the current supply agency clustering scheme, these two hubs are under Western cluster, which is one of the seven clusters consisting of three hubs (Jimma, Nekemte and Gambella). The agency hubs serve as outposts to distribute pharmaceuticals, chemical reagents, medical supplies, and equipment procured by the central office to localities on all corners of the country/region. They also gather pharmaceutical demand within their domain and communicate it with the head office to form an aggregate demand at national level. Hubs have been serving as key governmental structures for the implementation of integrated pharmaceutical and logistic services (IPLS) and distribution of essential health commodities for public health

For peer review only - http://bmjopen.bmj.com/site/about/guidelines.xhtml

facilities. They also provide supervisory, material support and capacity building to health facilities for strengthening and enforcing the implementation of IPLS and pharmacy services [21] (**diagram 1**). The pharmaceutical supply chain in Ethiopia consists of public, private actors and also partners where some of the manufacturing is done in-country. This system mainly comprises of manufacturers/suppliers, wholesalers and/or distributors to the health facilities as the main actors in the pharmaceutical supply chain map (**diagram 2**).

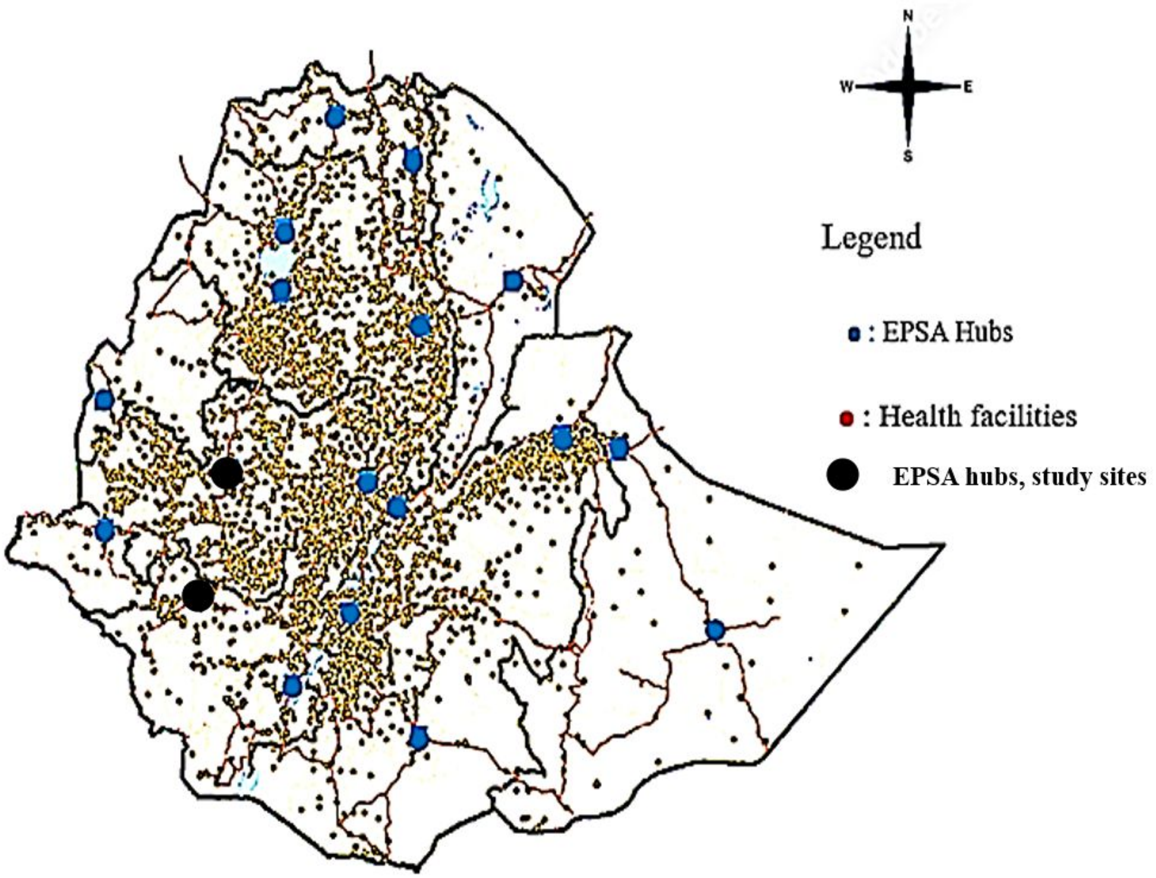

Diagram 1: Map of Ethiopia and location of Ethiopian pharmaceutical supply chain hubs (designed using Canva design; https://www.canva.com/design/DAFddjWPsAs/JOlltFCxb-GrvTh0y_Qt3w/edit)

For peer review only - http://bmjopen.bmj.com/site/about/guidelines.xhtml

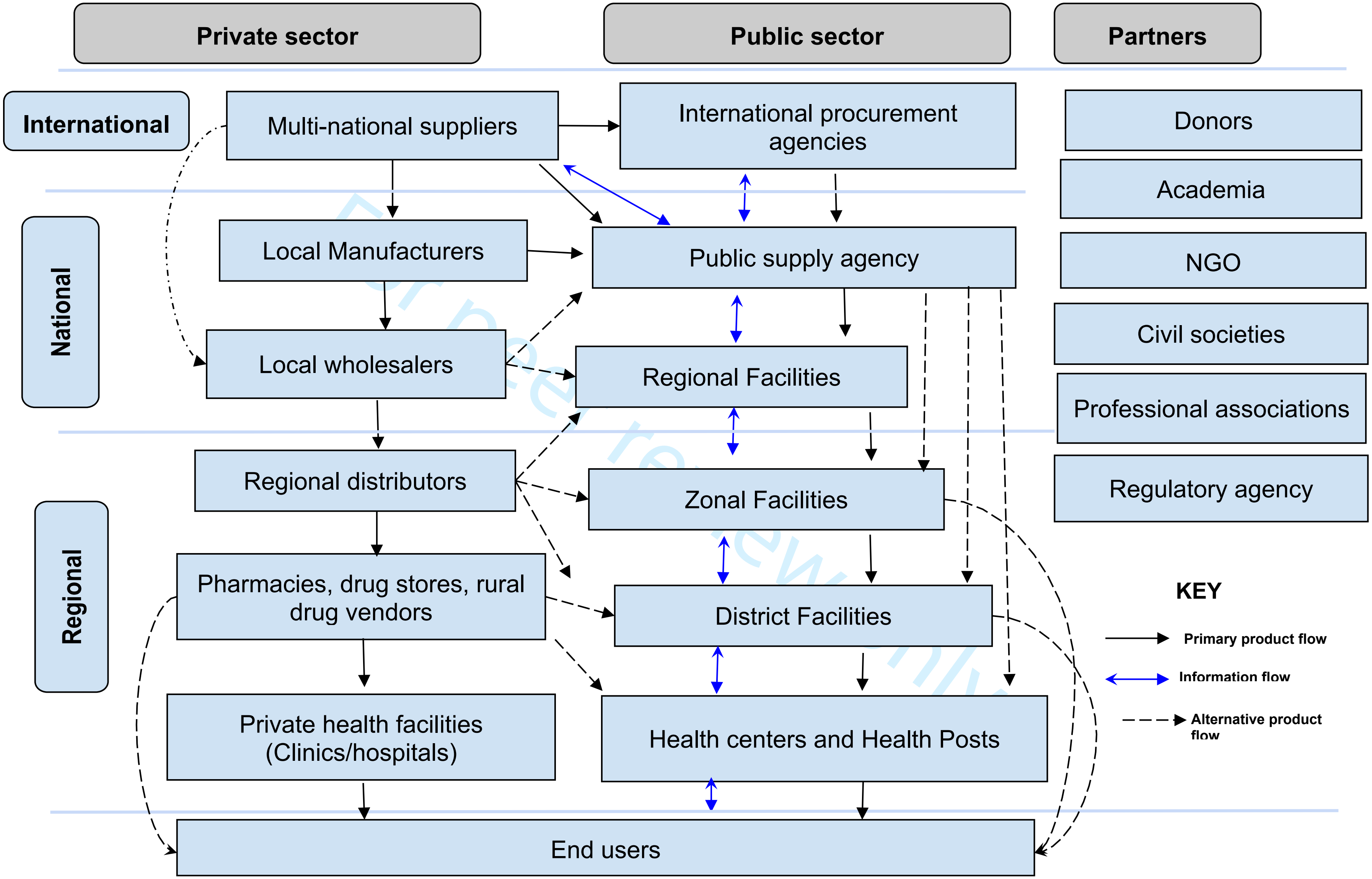

Diagram 2: Pharmaceutical supply chain map of Ethiopian pharmaceutical market (adapted from Management Sciences for Health 2012 [63]

For peer review only - http://bmjopen.bmj.com/site/about/guidelines.xhtml

2.2. Sample selection and time periods

Twenty-two (22) essential, generic medicines were selected from the essential medicines list of the country. Seventeen (17) were cardiovascular disease (CVD) drugs and the remaining five (5) antidiabetics. The products with lowest dosage strength and available formulation of the substance, which have a market authorization, were included. These are the most commonly prescribed medicines and dosages in the health facilities in Ethiopia. The LMIS records were used to track and compare product transactions data for eight months before COVID-19 (between 01 May and 31 December 2019) and for 12 months during pandemic time (between 01 January and 31 December 2020). This period was purposefully selected. In Ethiopia, the month of May/June marks the culmination of fiscal year activities by the government and the preparation of the New Year budget. Consequently, we have only eight months counting back from December 31st , 2019 (the outbreak of COVID-19) to obtain the most up-to-date information about the stock.

2.3. Data collection procedure

Based on the study objective, the modified data collection checklist from the standard tools [22, 23] was used to collect the relevant data. Closed-ended questions about the quantity of medicines ordered, received, distributed, and the stock situation on the day of data collection, were included on the observation checklist. Data was collected through a cross-sectional survey conducted over 12 months a period of time during the COVID-19 pandemic. The researchers visited pharmaceutical supply agencies and collected information on the availability of NCD medicines. They also recorded any shortages or stockouts that occurred during this period. A two-day training session was given to the data collectors regarding the study's objectives and how to collect the data. During the data collection process, there was close supervision by the investigators to ensure consistency and completeness of the data. The data was collected from the two public supply agency hubs.

2.4. Data processing and analysis

A descriptive frequency and percentage analysis was conducted to evaluate the line-item fill rate, product availability, and distribution trends before and after the COVID-19 pandemic. The following formulas were used to estimate the current product availability, stock out duration, and line-item fill rate of the selected medicines.

For peer review only - http://bmjopen.bmj.com/site/about/guidelines.xhtml

- $$\text{Medicine availability} = \frac{\text{Number of product available in a given period}}{\text{The number of product considered}} \times 100$$

- $$\text{Line–item fill rate} = \frac{\text{the amount (quantity) of item received}}{\text{The amount (quantity) of item ordered}} \times 100$$

- $$\text{Average stock–out duration} = \frac{\text{the number of days the product was not available}}{\text{The number of product reviewed}} \times 100$$

Based on the WHO's drug availability index, the analysis considered the availability of products that are ordered and received in the supply agencies as well as stock status on the day of data collection. A product is available if it is in stock in the health facility providing the service on the day of the visit or during the specified period. The following ranges were used to describe the medication availability status: less than 30% is very low; 30-49% is low; 50-80% is fairly high; and greater than 80% is very high [24, 25].

2.5. Patient and public involvement

It was not appropriate or possible to involve patients or the public in the design, conduct, reporting, or dissemination plans of our research.

For peer review only

For peer review only - http://bmjopen.bmj.com/site/about/guidelines.xhtml

3. Results

3.1. The line-item fill rate

The line-item fill rate indicates how often the EPSA hubs received at least 80% of the quantity ordered for a particular medicine. Prior to COVID-19, the overall average line-item fill rate for the selected products was 78%. It declined to 49% during the pandemic. For antidiabetics, the average line-item fill rate declined from 85% to 56%. For CVD drugs it went down from 76% to 46%. The overall line-fill rate dropped for all selected medicines during COVID-19 (**Figure 1 and 2)**.

3.2. Product availability and stock-out situation of selected basket medicines

During COVID-19, the overall mean (95% CI) of availability of the selected basket medicines was 43.3% (37.1- 49.5). However, it was 67.4 (62.2-72.6) before the outbreak. Comparing the mean availability of the two periods using a two-sample t-test, it showed a statistically significant difference (P<0.001). On the day of data collection, ten (10) of the twenty-two (46%) were out-of-stock.

Concerning the days out of stock, the mean (95% CI) was 11.7 (9.9- 13.5) days before COVID-19 outbreak, which was increased to 15.7 (13.2-18.2) days. This showed a statistically significant difference (P<0.001). Before the pandemic, the average stockout duration per month was 9 days for antidiabetics and 11 days for the CVD drugs. This increased to 13 days and 17 days/month, during the pandemic for antidiabetics and the CVD drugs, respectively. The medicines with the least supply were captopril 25mg tablets, metoprolol 25mg tablets, lovastatin 20mg tablets, and digoxin 0.25mg/ml injection. Lovastatin had the longest average stockout of 28 days, closely followed by digoxin injection at 27 days and captopril tablet at 26 days (resulting in WHO availability indices of 11%, 13 %, and 23%, respectively). The drop in the availability of specific medications ranged from 12% to 77% during the pandemic (**Table 1**).

For peer review only - http://bmjopen.bmj.com/site/about/guidelines.xhtml

Table 1: Product availability and average days out of stock for selected antidiabetics and CVD medications before and during COVID-19 pandemic

List of drug products	Pre-pandemic		During the pandemic		Decline in availability (%)
	Average days out of stock per month	Availability* (%)	Average days out of stock per month	Availability* (%)	
1. Insulin regular (soluble)	9.5	74.7	13.3	44.6	40
2. NPH insulin (suspension)	8	77.3	10.9	49	37
3. Insulin Isophane (biphasic)	8.5	75.2	12.1	43.4	42
4. Metformin 500 mg tablet	8.7	76.5	12.0	47.3	38
5. Glibenclamide 5mg tablet	10.3	70.6	12.3	56.9	19
6. Aspiring 81mg tablet	9.2	70.8	12.7	44	38
7. Enalapril 5mg tablet	11.0	68	14.4	47	31
8. Captopril 25mg tablet	16.7	56	26	23	59
9. Atenolol 50mg tablet	9.6	73.7	11.8	56.4	24
10. Propranolol 40 mg tablet	9.4	72.6	13.2	44	39
11. Metoprolol 25mg tablet	19	52	21.2	46	12
12. Frusemide 40mg tablet	9.3	71.5	12.4	47.7	33
13. Frusemide 10mg/ml	8.4	76	10	61.5	19
14. Hydrochlorothiazide 25mg tablet	10.8	68	16	35	49
15. Spironolactone 25mg tablet	9.6	74	11	63.5	14
16. Atorvastatin 20mg tablet	15	62	20	41.5	33
17. Lovastatin 20mg tablet	17	45	28	10.5	77
18. Methyldopa 250mg tablet	11.3	66.8	17.2	32	52
19. Amlodipine 5mg tablet	8.7	78	11.2	56	28
20. Nifedipine 20mg tablet	13.5	68.3	19.5	38	44
21. Digoxin 0.25mg tablet	9.6	74	12.4	52.5	29
22. Digoxin 0.25mg/ml (injection)	24	31	27	13	58

*Based on the WHO availability index [24, 25].

For peer review only - http://bmjopen.bmj.com/site/about/guidelines.xhtml

3.3. Unit price changes of selected antidiabetics and CVD medications

We were unable to assess the unit pricing changes of all medicines because of stockouts for the majority of the products. The prices were relatively stable for some medicines (where there was sufficient data to assess). Most of these products were from the local pharmaceutical industries. Yet for some of the products, there were dramatic price increases (**Figure 3**), such as for insulin products and methyldopa. Some of these products were imported and supplied by international pharmaceutical suppliers such as Novo Nordisk Pharmatech A/S. Likewise, the unit price for oral furosemide, amlodipine, and nifedipine increased starting from June 2020. During the study period, the value of the Ethiopian birr decreased by 38% compared to USD [26], dramatically increasing the price of imported medicines.

3.4. Selected medicine manufacturer/supplier

This paper uses the terms manufacturers and suppliers interchangeably. Sometimes facilities supply their stock from importer and/or wholesaler, sometimes directly from the manufacturer. For the selected NCD medicines, there were nineteen (19) manufacturers/suppliers over the period. During the eight months pre-pandemic, 192 orders were placed by the two agencies while during the 12 months of the pandemic 264 supply orders were placed. Ethiopian companies represented 50% and 53% of suppliers before and during COVID-19 pandemic, respectively. Similarly, European pharmaceutical industries were supplying 27% prior to COVID-19 and 31% during pandemic, meaning that about 84% NCD medicines at the two agencies were supplied either from Ethiopia or Europe during COVID-19 (**Table 2**).

For peer review only - http://bmjopen.bmj.com/site/about/guidelines.xhtml

Table 2 Lists of medicines manufacturer/supplier during & prior to the pandemic.

S. No	Manufacturers/suppliers name (country)	Percentage of supply orders covered	
		Before COVID-19	During COVID-19
1.	Amino AG (Switzerland)	4	8
2.	Addis Pharmaceutical Factory (Ethiopia)	8	13
3.	AstraZeneca (United Kingdom)	4	4
4.	Bliss GVS Pharma Limited (India)	-	3
5.	Brawn Laboratories Limited (India)	4	1
6.	Cadila Pharmaceuticals PLC (Ethiopia)	10	10
7.	Ciron Drugs & Pharmaceuticals PLC (India)	4	4
8.	East African Pharmaceuticals PLC (Ethiopia)	6	10
9.	EIPICO (Egypt)	4	4
10.	Epharm (Ethiopia)	5	1
11.	Gulf Pharmaceuticals Ltd (UAE)	4	4
12.	Humanwell Pharmaceutical PLC (Ethiopia)	4	1
13.	Intas Pharmaceuticals (India)	2	1
14.	Julphar Pharmaceuticals PLC (Ethiopia)	8	5
15.	Laboratoires Sterop (Belgium)	2	1
16.	Novo Nordisk Pharmatech A/S (Denmark)	4	5
17.	Remedica Ltd (Cyprus)	13	13
18.	Reyoung Pharmaceutical Co., Ltd (China)	4	1
19.	Sansheng Pharmaceutical PLC (Ethiopia)	9	13

Epharm: Ethiopian Pharmaceuticals Manufacturing share company EIPICO: Egyptian International Pharmaceutical Industries Company; UAE: United Arab Emirates

For peer review only - http://bmjopen.bmj.com/site/about/guidelines.xhtml

4. Discussion

Ensuring sustainable availability of essential medicines has been a persistent challenge in Ethiopia. The country has made progress in recent years, but the COVID-19 pandemic highlighted the need for further improvements. More must be done to strengthen the drug supply chain and ensure sustainable availability of essential medicines. This study assessed trends in the availability of selected medicines for chronic diseases at the public supply agencies over a long time-period. We evaluated the fill-item rate, trends, and product availability before and during the pandemic.

The results of the study showed that the COVID-19 pandemic significantly increased shortages of NCD medicines at pharmaceutical supply agencies. The researchers found that the stockout situation worsened during the pandemic, indicating challenges in the supply chain of essential medicines. Some of the challenges include disruptions in transportation and logistics, shortages of raw materials and active pharmaceutical ingredients, increased demand for certain medications, and workforce shortages due to illness or quarantine measures. Additionally, border closures and export restrictions have also impacted the availability of essential medicines in some regions. In response to the global burden of NCDs, the WHO has developed a Global Action Plan that includes target of 80% availability and affordability of essential medicines for the prevention and treatment of DM, CVD, and respiratory diseases [27]. Despite this strong recommendation, the availability of NCD medicines in Ethiopia falls below the recommended target. At the national level, the median availability of medicines for NCDs was below 50% with average stockout days of up to 27 days [28]. Similarly, the current study showed that the overall availability of selected medicines was 49% during the pandemic, down from 78% pre-pandemic. This includes wide variation, with lovastatin available only 11% according to the WHO availability index during the pandemic. This is consistent with studies from Kenya [29], Portugal [30] , Poland [31] and Congo and Cameroon [32]. This has led to increased difficulties for individuals with chronic conditions to access the medications they need to manage their health. The higher rates of stockouts mean that pharmacies and healthcare facilities are unable to meet the demand for these essential medicines, leaving patients without access to necessary treatments.

For peer review only - http://bmjopen.bmj.com/site/about/guidelines.xhtml

Not only were the medicines less available during COVID-19 but some also became more expensive, which is in line with several studies from countries like Bangladesh [41], Portugal [30], Saudi Arabia [42], India [43], Nigeria [44] and Namibia [45]. While the Ethiopian Birr decreased in value compared to the US Dollar by 38%, soluble insulin increased by more than 130% in price, which means exchange rates cannot alone explain the price increase. This suggests that other factors, such as supply chain disruptions or increased production costs, may have contributed to the price hike. Moreover, the changes in supplier dynamics on medication availability and affordability might contribute to the price changes during the pandemic. These findings highlight the need for further investigation into the underlying causes of medication price increases during crises. It is crucial to understand the specific factors influencing these price changes to develop effective strategies for mitigating their impact on individuals with chronic conditions. This study also suggests that while there were stable prices for certain medicines supplied by local pharmaceutical industries, there were price increases for insulin products and methyldopa, which were imported and supplied by international pharmaceutical suppliers. Factors such as supply chain disruptions, increased demand, and changes in production costs could contribute to these price fluctuations. It is important to further investigate the reasons behind these price increases, as they may have implications for medication affordability and accessibility.

Furthermore, this study also assessed the type of suppliers or manufacturers of selected drugs before and during the epidemic. The supply distribution did not vary substantially by region during and after COVID-19. For NCD medicines it appears that, at least these two public pharmaceutical supply agencies, are mainly supplied by Ethiopian and European companies. The large proportion of Ethiopian suppliers is beneficial, likely both in normal times and in pandemic, as the currency fluctuations should be smaller, although still present due to a likely dependence on imported active pharmaceutical ingredients (APIs). That local pharmaceutical industries were able to step up and meet the demand for these products demonstrates the importance of having strong and resilient local pharmaceutical industries that can provide essential healthcare products during times of crisis. This study highlights the importance of addressing supply chain disruptions during public health emergencies, such as the COVID-19 pandemic, to ensure the availability of

For peer review only - http://bmjopen.bmj.com/site/about/guidelines.xhtml

essential medicines for patients with NCDs. It emphasizes the need for interventions and strategies to strengthen the pharmaceutical supply chain and improve access to essential medicines in low-income countries. Overall, the COVID-19 pandemic has underscored the critical importance of maintaining a robust and resilient healthcare system that can effectively respond to emergencies while ensuring the availability and affordability of essential medicines for those who need them most.

Limitation of the study

To the best of the authors' knowledge, this is one of the first studies assessing supply situations of essential medicines during a pandemic at selected public pharmaceutical supply agencies. Yet, the results may not apply to all medicines. Other therapeutic areas may experience unique trends. The proximity to the capital city may allow for better distribution processes, whereas more remote areas may experience greater challenges. The study included only two facilities, which may have unique characteristics.

5. Conclusion

In summary, significant shortages of selected basket medicines used for the management of DM and CVD were observed at the two selected public pharmaceutical supply agencies. The pandemic has highlighted the importance of ensuring access to essential medications for patients with chronic conditions such as CVD and diabetes. Unit prices of some of the medicines dramatically increased, due to both currency fluctuations and other possible factors. The supply distribution did not vary substantially by region during and after COVID-19, with the two agencies mainly being supplied by Ethiopian and European companies. The findings of this study suggest that the COVID-19 pandemic has exacerbated existing medicines availability problems. Yet it also demonstrates a strong local provision of NCD medicines. Challenges facing these local providers should be further studied to understand how currency fluctuations, dependencies on imported APIs, and other factors influence their ability to supply the local population. It would also be useful to compare these findings with patient experiences to better understand how these stockouts impacted them and their health.

For peer review only - http://bmjopen.bmj.com/site/about/guidelines.xhtml

Abbreviations: APIs: Active Pharmaceutical Ingredients; CVD: Cardiovascular Diseases; COVID-19: coronavirus disease 2019; EPSA: Ethiopian Pharmaceutical Supply Agency; IRB: Institutional Review Board; SPSS: Statistical Package Software for Social Science; LMICs: low- and middle-income countries; IPLS: Integrated Pharmaceutical and Logistic Services; WHO: World Health Organization, LSAT: Logistics System Assessment Tool; IMAT: Inventory Management Assessment Tool; LIAT: Logistic Indicators Assessment Tool; NCDs: Non-communicable Diseases

Declarations

Ethics approval and consent to participate: Before commencing data collection, ethical approval was obtained from the Institutional Review Board (IRB) of the Institute of Health, Jimma University in Ethiopia (reference number JHRPG/1043/2020). Then, the selected public supply agencies were communicated with a formal letter from the health research and innovation office of Jimma University. The study was conducted after getting permission from the manager of EPSA.

Consent for publication: Not required.

Availability of data and materials: This study included all pertinent data. Additional information could be obtained from the corresponding author upon reasonable request.

Competing interests: None declared.

Funding: This work was supported by the Global Health and vaccination research programme
(GLOBVAC), which is the programme of the Research Council of Norway.

Authors' contribution: ZM, TSM, GTT, MM, CÅ, and MJ designed the study. ZM and TSM drafted the first version of the manuscript. ZM, TSM, CÅ, and MJ discussed the analytic strategy. ZM, TSM, and GTT did the data curation and performed the analysis. All authors reviewed the final paper and made creative and substantial contributions, approved the final version, and consented to publication.

Acknowledgements: We would like to extend our appreciation and thank our data collectors and study participants. We also extend our sincere gratitude to health care

For peer review only - http://bmjopen.bmj.com/site/about/guidelines.xhtml

professionals, and managers at supply agency hubs for providing us with necessary data/information, and for their kind cooperation, and assistance throughout the data collection process. Additionally, we would like to express our heartfelt gratitude to the Global Health and Vaccination Research Programme (GLOBVAC) for financially supporting this research under the auspices of the MIA (Measures for Improved Availability of Medicines and Vaccines (The MIA Project | BI). Finally, our acknowledgment extends to the Institute of Health of Jimma University for supporting us in the ethical approval process and in the official communication of respective offices and health facilities.

References

1. World Health Organization (WHO, 2006), Hogerzeil, H.V., *Essential medicines and human rights: what can they learn from each other?* Bulletin of the World Health Organization, 2006. **84**: p. 371-375.
2. De Weerdt, E., et al., *Toward a European definition for a drug shortage: a qualitative study.* Frontiers in pharmacology, 2015. **6**: p. 253.
3. Wagenaar, B.H., et al., *Stock-outs of essential health products in M ozambique–longitudinal analyses from 2011 to 2013.* Tropical Medicine & International Health, 2014. **19**(7): p. 791-801.
4. Wild, L. and D. Cammack, *The supply and distribution of essential medicines in Malawi.* 2013.
5. Kohler, J.C., et al., *Corruption in the pharmaceutical sector: diagnosing the challenges.* 2016.
6. Martinez, M.G., J.C. Kohler, and H. McAlister, *Corruption in the pharmaceutical sector*, in *The Handbook of Business and Corruption*. 2017, Emerald Publishing Limited. p. 329-361.
7. Besancon, L. and B. Chaar, *Report of the International Summit on medicines shortage.* Toronto, Canada: International Pharmaceutical Federation, 2013.
8. Morrison, A., *Drug supply disruptions [environmental scan issue 17].* Ottawa: Canadian Agency for Drugs and Technologies in Health. Available online at: https://www. cadth. ca/drug-supply-disruptions, 2011.
9. World Health Organization (WHO, 2017)., *Access to medicines: making market forces serve the poor.* Geneva, Switzerland, 2017.
10. Ahlqvist, V., et al., *Supply chain risk management strategies in normal and abnormal times: policymakers' role in reducing generic medicine shortages.* International Journal of Physical Distribution & Logistics Management, 2023. **53**(2): p. 206-230.
11. Magableh, G.M., *Supply chains and the COVID-19 pandemic: A comprehensive framework.* European Management Review, 2021. **18**(3): p. 363-382.
12. Yu, Z., et al., *Disruption in global supply chain and socio-economic shocks: a lesson from COVID-19 for sustainable production and consumption.* Operations Management Research, 2021: p. 1-16.

For peer review only - http://bmjopen.bmj.com/site/about/guidelines.xhtml

13. Ziavrou, K.S., S. Noguera, and V.A. Boumba, *Trends in counterfeit drugs and pharmaceuticals before and during COVID-19 pandemic.* Forensic Science International, 2022: p. 111382.
14. Noguera, S. and K. Ziavrou, *With the world in turmoil, transnational organized crime sees opportunity in counterfeit pharmaceuticals and medical supplies during the COVID-19 pandemic.* Perspectives, 2020. **12**.
15. Ozili, P.K. and T. Arun, *Spillover of COVID-19: impact on the Global Economy.* Available at SSRN 3562570, 2020.
16. Bookwalter, C.M., *Drug shortages amid the COVID-19 pandemic.* Us Pharm, 2021. **46**(2): p. 25-8.
17. World Health Organization (WHO, 2023),, *COVID-19 pandemic significantly impacted access to medicines for noncommunicable diseases; https://www.who.int/news/item/22-03-2023-covid-19-pandemic-significantly-impacted-access-to-medicines-for-noncommunicable-diseases; access date 20 April 2023.* March 2023.
18. World Health Organization (WHO, 2023), *Access to NCD medicines: emergent issues during the COVID-19 pandemic and key structural factors.* 2023.
19. Sen-Crowe, B., M. McKenney, and A. Elkbuli, *Medication shortages during the COVID-19 pandemic: Saving more than COVID lives.* The American journal of emergency medicine, 2021. **45**: p. 557.
20. Mekonnen, Z., et al., The knock-on effects of COVID-19 pandemic on the supply and availability of generic medicines in Ethiopia: mixed methods study. BMC Health Services Research, 2023. 23(1): p. 1-16.
21. Agency, E.P.S., *Info Hubs - Ethiopian Pharmaceuticals Supply Agency(EPSA); https://epss.gov.et/info-hubs/; Accessed date: May, 22, 2022.* 2021.
22. USAID and T.O. DELIVER PROJECT, *Logistics Indicators Assessment Tool (LIAT).* 2008, USAID Arlington, VA.
23. USAID and T.O. DELIVER PROJECT, *Logistics System Assessment Tool (LSAT).* 2009, USAID| DELIVER PROJECT, Task Order 1, Arlington.
24. World Health Organization (WHO, 2008),, *Measuring medicine prices, availability, affordability and price components*. 2008, World Health Organization.
25. World Health Organization (WHO, 2017), *Technical definitions of shortages and Stockouts of medicines and vaccines.* Geneva: World Health Organization, 2017.
26. Exchange-Rates.org, *US Dollar (USD) To Ethiopian Birr (ETB) Exchange Rate History for December 31, 2019; https://www.exchange-rates.org/exchange-rate-history/usd-etb-2019-12-31. accessed date: 20 May 2023.* 2023.
27. World Health Organization (WHO, 2013), *Global action plan for the prevention and control of noncommunicable diseases 2013-2020*. 2013: World Health Organization.
28. ETHiopian Ministry of Health, FDRE, 2003 *Assessment of the pharmaceutical sector in Ethiopia*. 2003, Addis Ababa, Ethiopia.: Ministry of Health.
29. Kiragu, Z.W., et al., *Household access to non-communicable disease medicines during universal health care roll-out in Kenya: A time series analysis.* PloS one, 2022. **17**(4): p. e0266715.
30. Romano, S., et al., *Time-trend analysis of medicine sales and shortages during COVID-19 outbreak: Data from community pharmacies.* Research in Social and Administrative Pharmacy, 2021. **17**(1): p. 1876-1881.

For peer review only - http://bmjopen.bmj.com/site/about/guidelines.xhtml

31. Nowak, B.M., et al., *Availability of Cardiodiabetological Drugs in Poland during the First Year of COVID-19 Pandemic: Retrospective Study.* BioMed, 2022. **2**(1): p. 117-126.
32. Schäfermann, S., et al., *Availability, prices and affordability of selected antibiotics and medicines against non-communicable diseases in western Cameroon and northeast DR Congo.* Plos one, 2020. **15**(1): p. e0227515.
33. Attaei, M.W., et al., *Availability and affordability of blood pressure-lowering medicines and the effect on blood pressure control in high-income, middle-income, and low-income countries: an analysis of the PURE study data.* The Lancet Public Health, 2017. **2**(9): p. e411-e419.
34. Ewen, M., et al., *Baseline assessment of WHO's target for both availability and affordability of essential medicines to treat non-communicable diseases.* PloS one, 2017. **12**(2): p. e0171284.
35. Cameron, A., et al., *Medicine prices, availability, and affordability in 36 developing and middle-income countries: a secondary analysis.* The lancet, 2009. **373**(9659): p. 240-249.
36. Mendis, S., et al., *The availability and affordability of selected essential medicines for chronic diseases in six low-and middle-income countries.* Bulletin of the world health organization, 2007. **85**(4): p. 279-288.
37. van Mourik, M.S., et al., *Availability, price and affordability of cardiovascular medicines: a comparison across 36 countries using WHO/HAI data.* BMC Cardiovascular disorders, 2010. **10**(1): p. 1-9.
38. Husain, M.J., et al., *Access to cardiovascular disease and hypertension medicines in developing countries: an analysis of essential medicine lists, price, availability, and affordability.* Journal of the American Heart Association, 2020. **9**(9): p. e015302.
39. Das, M., *Lebanon faces critical shortage of drugs.* The Lancet Oncology, 2021. **22**(8): p. 1063.
40. (ADA), A.D.A., *NEW DATA ALERT: COVID-19 Brings Crisis of Access for Millions Living with Diabetes; https://diabetes.org/newsroom/press-releases/COVID-19-brings-crisis-of-access-for-millions-living-with-diabetes Acceessed date 15 January 2023.* December 23, 2020.
41. Haque, M., et al., *Availability and price changes of potential medicines and equipment for the prevention and treatment of COVID-19 among pharmacy and drug stores in Bangladesh; findings and implications.* Bangladesh Journal of Medical Science, 2020: p. 36-S 50.
42. Aljadeed, R., et al. *The impact of COVID-19 on essential medicines and personal protective equipment availability and prices in Saudi Arabia*. in *Healthcare*. 2021. MDPI.
43. Haque, M., et al., *Utilisation, availability and price changes of medicines and protection equipment for COVID-19 among selected regions in India: findings and implications.* Frontiers in pharmacology, 2021. **11**: p. 582154.
44. Awucha, N.E., et al., *Impact of the COVID-19 pandemic on consumers' access to essential medicines in Nigeria.* The American journal of tropical medicine and hygiene, 2020. **103**(4): p. 1630.

For peer review only - http://bmjopen.bmj.com/site/about/guidelines.xhtml

45. Tirivangani, T., et al., *Impact of COVID-19 pandemic on pharmaceutical systems and supply chain–a phenomenological study.* Exploratory Research in clinical and social pharmacy, 2021. **2**: p. 100037.
46. Khader, M.A., T. Jabeen, and R. Namoju, *A cross sectional study reveals severe disruption in glycemic control in people with diabetes during and after lockdown in India.* Diabetes & Metabolic Syndrome: Clinical Research & Reviews, 2020. **14**(6): p. 1579-1584.
47. Singh, K., et al., *Health, psychosocial, and economic impacts of the COVID-19 pandemic on people with chronic conditions in India: a mixed methods study.* BMC public health, 2021. **21**: p. 1-15.
48. De Vet, J.M., et al., *Impacts of the COVID-19 pandemic on EU industries*. 2021: European Parliament Strasbourg, France.
49. Bucher, A. and G.B. Wolff, *Increasing the global supply of essential medical supplies: Time for Europe to step up its global leadership.* Bruegel-Blogs, 2021: p. NA-NA.
50. Gereffi, G., *What does the COVID-19 pandemic teach us about global value chains? The case of medical supplies.* Journal of International Business Policy, 2020. **3**: p. 287-301.
51. Zamiela, C., N.U.I. Hossain, and R. Jaradat, *Enablers of resilience in the healthcare supply chain: A case study of US healthcare industry during COVID-19 pandemic.* Research in Transportation Economics, 2022. **93**: p. 101174.
52. Express, F., *J.U. Haroon, Pharma Industry Growth Halves in 2020; https://www.thefinancialexpress.com.bd/trade/pharma-industry-growth-halves-in-2020-1610159516 Accessed date 03 January, 2023.* 2020.
53. Elsafty, A. and M. Osman, *The Impact of COVID-19 on the Efficiency of Packing Lines in Pharmaceutical Manufacturing Sites in Egypt.* International Journal of Business and Management, 2021. **16**(7): p. 57-72.
54. Joshi, A., P. Bhaskar, and P.K. Gupta, *Indian economy amid COVID-19 lockdown: A prespective.* J. Pure Appl. Microbiol, 2020. **14**: p. 957-961.
55. Purwanto, A., *The Covid-19 pandemic impact on industries performance: an explorative study of Indonesian companies.* Journal of Critical Reviews, 2020.
56. Abdelhadi, A. and E. Akkartal, *The Impact Of COVID-19 on Global Supply Chain.*
57. Lozano-Diez, J., J. Marmolejo-Saucedo, and R. Rodriguez-Aguilar, *designing a resilient supply chain: An approach to reduce drug shortages in epidemic outbreaks.* EAI Endorsed Transactions on Pervasive Health and Technology, 2020. **6**(21): p. e2.
58. Adebisi, Y., A. Jumoke, and O. Carolyn, *Coronavirus disease-19 and access to medicines in Africa.* International Journal of Health & Allied Sciences, 2020(5): p. 120-120.
59. Wirtz, V.J., et al., *Essential medicines for universal health coverage.* The Lancet, 2017. **389**(10067): p. 403-476.
60. Suleman, S., et al., *Pharmaceutical regulatory framework in Ethiopia: a critical evaluation of its legal basis and implementation.* Ethiopian journal of health sciences, 2016. **26**(3): p. 259-276.
61. Ewen, M., et al., *Prices and availability of locally produced and imported medicines in Ethiopia and Tanzania.* Journal of pharmaceutical policy and practice, 2017. **10**(1): p. 1-9.

For peer review only - http://bmjopen.bmj.com/site/about/guidelines.xhtml

62. Marew, T., et al., *Trends and Challenges in Access to Essential Medicines in Ethiopia and the Contributions of Local Pharmaceutical Production.* Ethiopian Journal of Health Sciences, 2022. **32**(5): p. 1027-1042
63. Management Science for Health (2012); Chapter 22-Managing distribution; available from https://msh.org/wp-content/uploads/2013/04/mds3-ch22-distribution-mar2012.pdf: accessed date 4 December 2023.

For peer review only

For peer review only - http://bmjopen.bmj.com/site/about/guidelines.xhtml

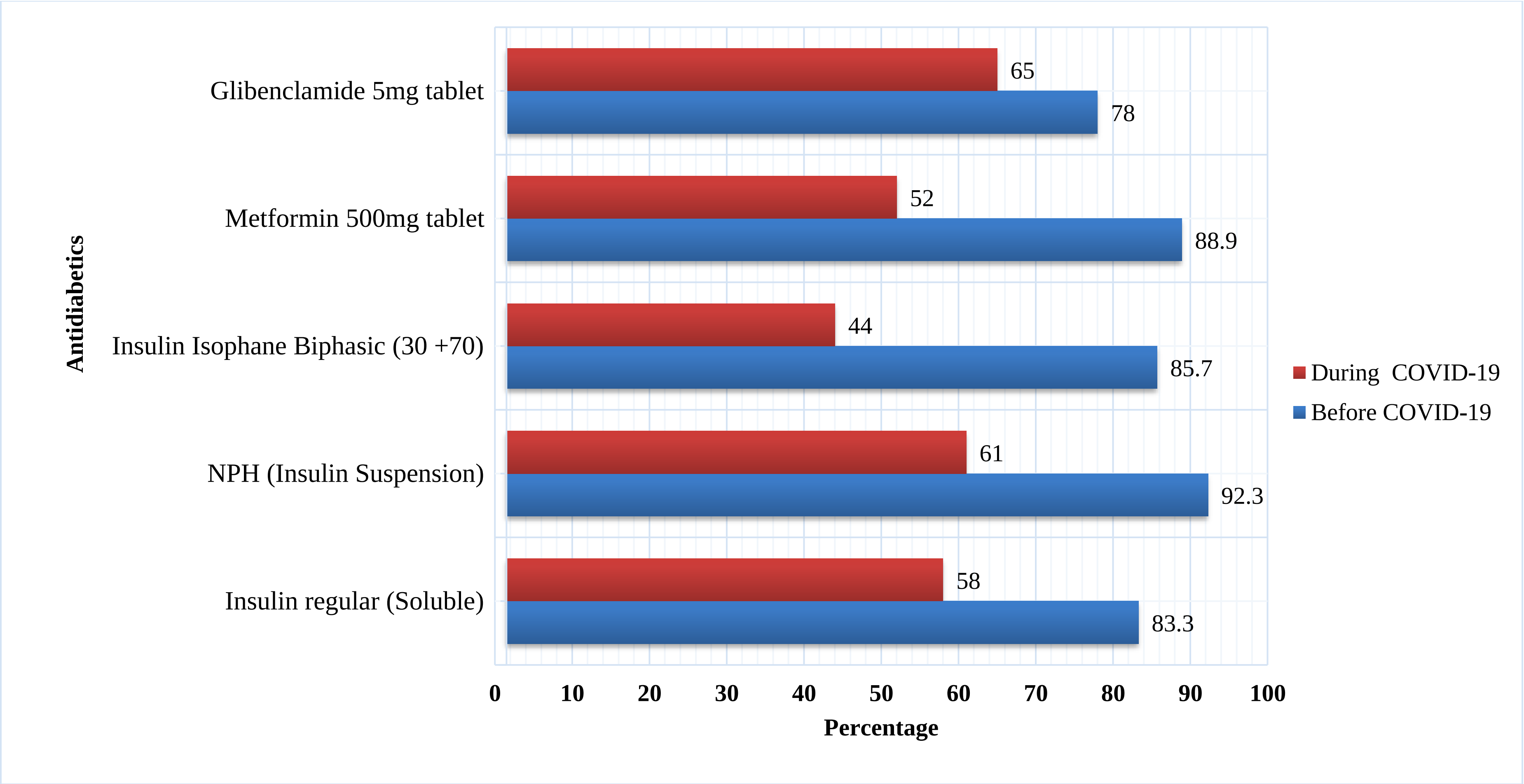

Figure 1: The average line-item fill rate for selected antidiabetics before and during COVID-19

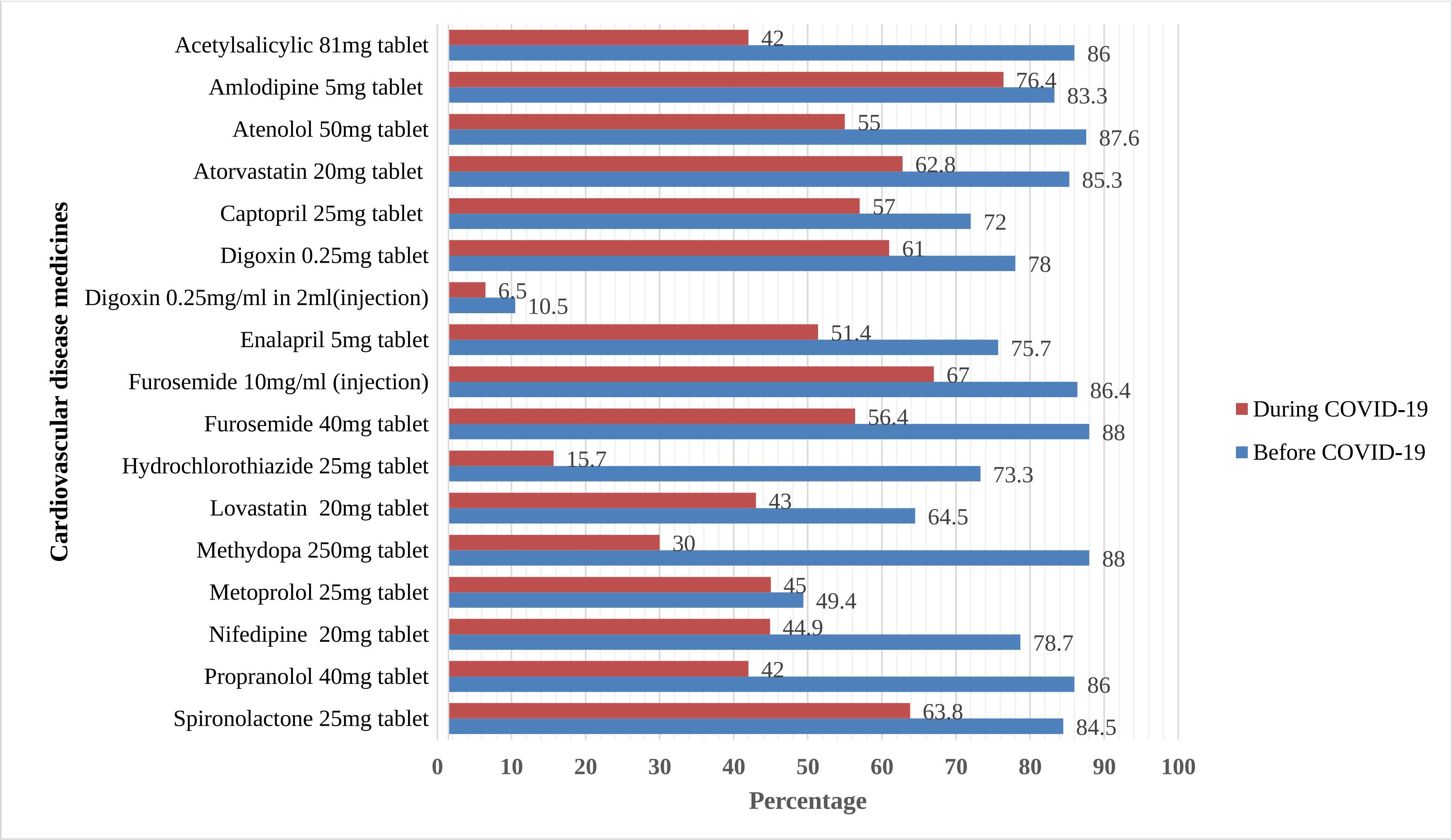

Figure 2: The average line-item fill rate for selected CVD medications before and during COVID-19.

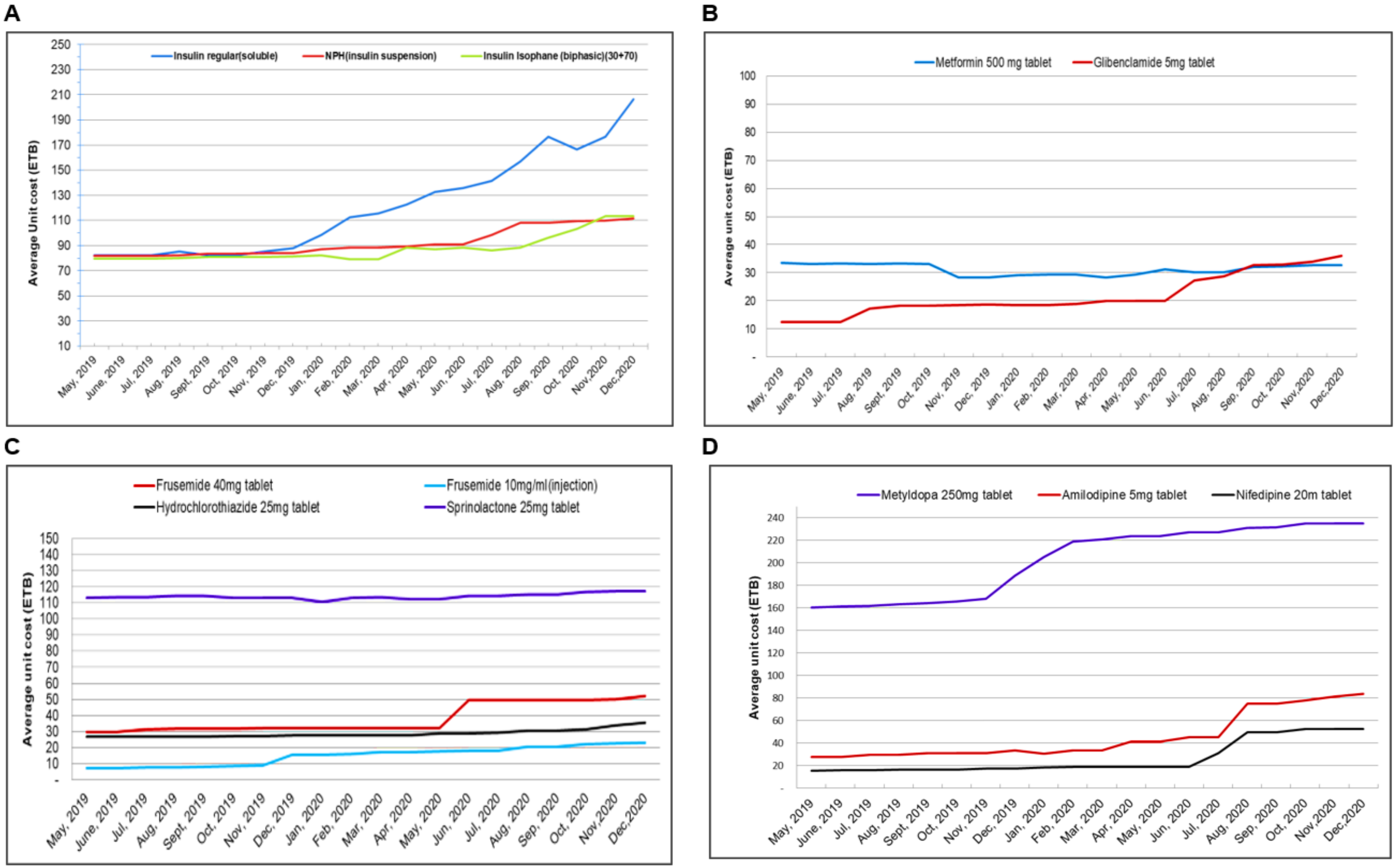

Figure 3: Trends of selected antidiabetics (A, B) and CVD medicines (C, D) unit prices at public pharmaceutical supply agency hub

STROBE Statement—Checklist of items that should be included in reports of ***cross-sectional studies***

	Item No	Recommendation	Page No
**Title and abstract**	1	(*a*) Indicate the study's design with a commonly used term in the title or the abstract	1
		(*b*) Provide in the abstract an informative and balanced summary of what was done and what was found	3
**Introduction**			
Background/rationale	2	Explain the scientific background and rationale for the investigation being reported	4
Objectives	3	State specific objectives, including any prespecified hypotheses	5
**Methods**			
Study design	4	Present key elements of study design early in the paper	5
Setting	5	Describe the setting, locations, and relevant dates, including periods of recruitment, exposure, follow-up, and data collection	5
Participants	6	(*a*) Give the eligibility criteria, and the sources and methods of selection of participants	5-6
Variables	7	Clearly define all outcomes, exposures, predictors, potential confounders, and effect modifiers. Give diagnostic criteria, if applicable	5-7
Data sources/ measurement	8*	For each variable of interest, give sources of data and details of methods of assessment (measurement). Describe comparability of assessment methods if there is more than one group	*8*
Bias	9	Describe any efforts to address potential sources of bias	NA
Study size	10	Explain how the study size was arrived at	8
Quantitative variables	11	Explain how quantitative variables were handled in the analyses. If applicable, describe which groupings were chosen and why	8-9
Statistical methods	12	(*a*) Describe all statistical methods, including those used to control for confounding	8-9
		(*b*) Describe any methods used to examine subgroups and interactions	8-9
		(*c*) Explain how missing data were addressed	
		(*d*) If applicable, describe analytical methods taking account of sampling strategy	NA
		(*e*) Describe any sensitivity analyses	NA
**Results**			
Participants	13*	(a) Report numbers of individuals at each stage of study—eg numbers potentially eligible, examined for eligibility, confirmed eligible, included in the study, completing follow-up, and analysed	10
		(b) Give reasons for non-participation at each stage	10
		(c) Consider use of a flow diagram	NA
Descriptive data	14*	(a) Give characteristics of study participants (eg demographic, clinical, social) and information on exposures and potential confounders	10
		(b) Indicate number of participants with missing data for each variable of interest	NA
Outcome data	15*	Report numbers of outcome events or summary measures	
Main results	16	(*a*) Give unadjusted estimates and, if applicable, confounder-adjusted estimates and their precision (eg, 95% confidence interval). Make clear which confounders were adjusted for and why they were included	10-17

For peer review only

		(*b*) Report category boundaries when continuous variables were categorized	NA
		(*c*) If relevant, consider translating estimates of relative risk into absolute risk for a meaningful time period	NA
Other analyses	17	Report other analyses done—eg analyses of subgroups and interactions, and sensitivity analyses	NA
**Discussion**			
Key results	18	Summarise key results with reference to study objectives	18-20
Limitations	19	Discuss limitations of the study, taking into account sources of potential bias or imprecision. Discuss both direction and magnitude of any potential bias	20
Interpretation	20	Give a cautious overall interpretation of results considering objectives, limitations, multiplicity of analyses, results from similar studies, and other relevant evidence	20
Generalisability	21	Discuss the generalisability (external validity) of the study results	20
**Other information**			
Funding	22	Give the source of funding and the role of the funders for the present study and, if applicable, for the original study on which the present article is based	21

*Give information separately for exposed and unexposed groups.

Note: An Explanation and Elaboration article discusses each checklist item and gives methodological background and published examples of transparent reporting. The STROBE checklist is best used in conjunction with this article (freely available on the Web sites of PLoS Medicine at http://www.plosmedicine.org/, Annals of Internal Medicine at http://www.annals.org/, and Epidemiology at http://www.epidem.com/). Information on the STROBE Initiative is available at www.strobe-statement.org.

For peer review only

For peer review only - http://bmjopen.bmj.com/site/about/guidelines.xhtml

BMJ Open

Availability of essential, generic medicines before and during COVID-19 at selected public pharmaceutical supply agencies in Ethiopia: Comparative cross-sectional study

Journal:	*BMJ Open*
Manuscript ID	bmjopen-2023-077545.R2
Article Type:	Original research
Date Submitted by the Author:	23-Dec-2023
Complete List of Authors:	Melaku , Tsegaye; Jimma University, Institute of Health<br>Mekonnen, Zeleke; Jimma University College of Public Health and Medical Sciences, School of Medical Laboratory Sciences;<br>Terefe Tucho, Gudina; Jimma University<br>Mecha, Mohammed ; Jimma University<br>Årdal, Christine ; Norwegian Institute of Public Health<br>Jahre, Marianne ; BI Norwegian Business School
**Primary Subject Heading**:	Public health
Secondary Subject Heading:	Cardiovascular medicine, Global health, Health services research, Pharmacology and therapeutics, Patient-centred medicine
Keywords:	Health Services Accessibility, HEALTH SERVICES ADMINISTRATION & MANAGEMENT, COVID-19

SCHOLARONE™
Manuscripts

I, the Submitting Author has the right to grant and does grant on behalf of all authors of the Work (as defined in the below author licence), an exclusive licence and/or a non-exclusive licence for contributions from authors who are: i) UK Crown employees; ii) where BMJ has agreed a CC-BY licence shall apply, and/or iii) in accordance with the terms applicable for US Federal Government officers or employees acting as part of their official duties; on a worldwide, perpetual, irrevocable, royalty-free basis to BMJ Publishing Group Ltd ("BMJ") its licensees and where the relevant Journal is co-owned by BMJ to the co-owners of the Journal, to publish the Work in this journal and any other BMJ products and to exploit all rights, as set out in our licence.

The Submitting Author accepts and understands that any supply made under these terms is made by BMJ to the Submitting Author unless you are acting as an employee on behalf of your employer or a postgraduate student of an affiliated institution which is paying any applicable article publishing charge ("APC") for Open Access articles. Where the Submitting Author wishes to make the Work available on an Open Access basis (and intends to pay the relevant APC), the terms of reuse of such Open Access shall be governed by a Creative Commons licence – details of these licences and which Creative Commons licence will apply to this Work are set out in our licence referred to above.

Other than as permitted in any relevant BMJ Author's Self Archiving Policies, I confirm this Work has not been accepted for publication elsewhere, is not being considered for publication elsewhere and does not duplicate material already published. I confirm all authors consent to publication of this Work and authorise the granting of this licence.

For peer review only

Availability of essential, generic medicines before and during COVID-19 at selected public pharmaceutical supply agencies in Ethiopia: Comparative cross-sectional study

[1]Tsegaye Melaku¥*, [1]Zeleke Mekonnen¥, [1]Gudina Terefe Tucho, [1]Mohammed Mecha, [2]Christine Årdal, [3]Marianne Jahre

[1]Institute of Health, Jimma University, Jimma, Oromia, Ethiopia

[2]Norwegian Institute of Public Health, Oslo, Norway

[3]BI Norwegian Business School, Oslo, Norway

*Corresponding author ¥two authors equally contributed to the work

Address of correspondence

- Email: tsegish.melaku@gmail.com
- Phone: +251913765609

For peer review only

Abstract

Objectives Lockdowns and border closures impacted medicines availability during the COVID-19 pandemic. This study aimed to assess the availability of essential, generic medicines for chronic diseases at public pharmaceutical supply agencies in Ethiopia.

Design Comparative cross-sectional study

Setting The availability of essential, generic medicines for chronic diseases was assessed at two public pharmaceutical supply agency hubs.

Participants The current study included public supply agency hub managers, warehouse managers and forecasting officers at the study setting.

Outcomes The assessment encompassed the availability of chronic medicines on the day of data collection, as well as records spanning eight months before the outbreak and one year during the pandemic. A total of twenty-two medicines were selected based on their inclusion in the national essential drug list for public health facilities, including seventeen medicines for cardiovascular disease and five for diabetes mellitus.

Results The results of the study indicate that the mean availability of the selected basket medicines was 43.3% (95% CI: 37.1-49.5) during COVID-19, which was significantly lower than the availability of 67.4% (95% CI: 62.2-72.6) before the outbreak ($P<0.001$). Prior to COVID-19, the overall average line-item fill rate for the selected products was 78%, but it dropped to 49% during the pandemic. Furthermore, the mean number of days out of stock per month was 11.7 (95% CI: 9.9-13.5) before the outbreak of COVID-19, which significantly increased to 15.7 (95% CI: 13.2-18.2) during the pandemic, indicating a statistically significant difference ($P<0.001$). Although the prices for some drugs remained relatively stable, there were significant price hikes for some products. For example, the unit price of insulin increased by more than 130%.

Conclusion The COVID-19 pandemic worsened the availability of essential chronic medicines, including higher rates of stockouts and unit price hikes for some products in the study setting. The study's findings imply that the COVID-19 pandemic has aggravated already-existing medicine availability issues. Efforts should be made to develop contingency plans and establish mechanisms to monitor medicine availability and pricing during such crises.

For peer review only - http://bmjopen.bmj.com/site/about/guidelines.xhtml

Keywords: Availability, COVID—19, Essential medicines, Ethiopia, Manufacturer, Supplier, Stock out

For peer review only

For peer review only - http://bmjopen.bmj.com/site/about/guidelines.xhtml

Strengths and limitations of this study

- Provides a comparative assessment of selected generic medicine's availability used for chronic diseases before and during COVID-19.
- The quality of the data in our study is high as it utilizes a standardized methodological tool recommended by World Health Organization.
- Limited geographic location will affect the study representativeness.

1. Introduction

Medicines are vital elements to healthcare, and access to medicines is a fundamental human right [1]. The World Health Organization (WHO) defines essential medicines as those which "satisfy the population's priority health care needs" [2]. Drug shortages are commonplace and present significant problems in all countries, including developed nations. The causes of drug shortages are complex and multifaceted, and addressing them requires collaborative effort from various stakeholders. The majority of the developing countries, mostly in sub-Saharan Africa, lack consistent availability and access to essential health products [3, 4]. This is due to various factors including inadequate infrastructure, insufficient funding, currency fluctuations, and more system related factors like poor governance and corruptions [5, 6][7-10].

Although various emergency situations and supply contamination have shut down key manufacturing sites in the past, the COVID-19 pandemic was the first sustained shock event to affect all parts of the global pharmaceutical supply chain [11, 12]. During the pandemic, countries across the world went into lockdowns, shutting down or reducing transport within and between them. This affected the manufacturing, supply, and distribution of medicines, leading to constraints in the medicines supply chain. A general shortage of various types of pharmaceuticals at supply and retail levels has been recorded in many nations, along with price rises and increases in falsified products [13, 14]. The impact of the COVID-19 pandemic on the global medical supply chain has been exacerbated by core weaknesses in relation to essential medicines production and procurement [15, 16].

For peer review only - http://bmjopen.bmj.com/site/about/guidelines.xhtml

Drug shortages caused by the COVID-19 outbreak were mostly fueled by spikes in demand for certain kinds of medications such as medicines used for the management of non-communicable diseases (NCDs) [16-18]. People with NCDs need uninterrupted, reliable access to quality-assured and affordable medicines and health products. During the pandemic service delivery points experienced shortages of medications used to treat cardiovascular disease (CVD) and diabetes mellitus (DM) [18, 19]. Most low-income governments, especially those in sub-Saharan Africa, opt for a distribution strategy where they purchase medicines and deliver them to health facilities via a publicly run Central Medical Store (CMS) and a government-owned transport fleet. In recent research, which assessed the availability of the same basket of NCD medicines at the health facility level showed that COVID-19 significantly increased shortages of these products and worsened the stockout situation at the health facilities [20.]. Expanding upon its antecedent, the present manuscript delineates and scrutinizes the impact of the COVID-19 pandemic on the supply chain dynamics of non-communicable disease (NCD) medications at the level of pharmaceutical supply agencies, immediately preceding their distribution to healthcare facilities.

2. Methods

2.1. Study setting

The study was conducted at two Ethiopian pharmaceutical supply agency (EPSA) hubs (Nekemte and Jimma) in southwestern Ethiopia. Both hubs, Nekemte and Jimma, are approximately 300 kilometers and 350 kilometers from Addis Ababa, respectively and about 200 kilometers from each other. According to the current supply agency clustering scheme, these two hubs are under Western cluster, which is one of the seven clusters consisting of three hubs (Jimma, Nekemte and Gambella). The agency hubs serve as outposts to distribute pharmaceuticals, chemical reagents, medical supplies, and equipment procured by the central office to localities on all corners of the country/region. They also gather pharmaceutical demand within their domain and communicate it with the head office to form an aggregate demand at national level. Hubs have been serving as key governmental structures for the implementation of integrated pharmaceutical and logistic services (IPLS) and distribution of essential health commodities for public health

For peer review only - http://bmjopen.bmj.com/site/about/guidelines.xhtml

facilities. They also provide supervisory, material support and capacity building to health facilities for strengthening and enforcing the implementation of IPLS and pharmacy services [21]. The pharmaceutical supply chain in Ethiopia consists of public, private actors and also partners where some of the manufacturing is done in-country. This system mainly comprises of manufacturers/suppliers, wholesalers and/or distributors to the health facilities as the main actors in the pharmaceutical supply chain map (**diagram 1**).

For peer review only

For peer review only - http://bmjopen.bmj.com/site/about/guidelines.xhtml

Diagram 1: Pharmaceutical supply chain map of Ethiopian pharmaceutical market (adapted from Management Sciences for Health 2012 [22]

For peer review only

For peer review only - http://bmjopen.bmj.com/site/about/guidelines.xhtml

2.2. Sample selection and time periods

Twenty-two (22) essential, generic medicines were selected from the essential medicines list of the country. Seventeen (17) were cardiovascular disease (CVD) drugs and the remaining five (5) antidiabetics. The products with lowest dosage strength and available formulation of the substance, which have a market authorization, were included. These are the most commonly prescribed medicines and dosages in the health facilities in Ethiopia. The LMIS records were used to track and compare product transactions data for eight months before COVID-19 (between 01 May and 31 December 2019) and for 12 months during pandemic time (between 01 January and 31 December 2020). This period was purposefully selected. In Ethiopia, the month of May/June marks the culmination of fiscal year activities by the government and the preparation of the New Year budget. Consequently, we have only eight months counting back from December 31st , 2019 (the outbreak of COVID-19) to obtain the most up-to-date information about the stock.

2.3. Data collection procedure

Based on the study objective, the modified data collection checklist from the standard tools [23, 24] was used to collect the relevant data. Closed-ended questions about the quantity of medicines ordered, received, distributed, and the stock situation on the day of data collection, were included on the observation checklist. Data was collected through a cross-sectional survey conducted over 12 months a period of time during the COVID-19 pandemic. The researchers visited pharmaceutical supply agencies and collected information on the availability of NCD medicines. They also recorded any shortages or stockouts that occurred during this period. A two-day training session was given to the data collectors regarding the study's objectives and how to collect the data. During the data collection process, there was close supervision by the investigators to ensure consistency and completeness of the data. The data was collected from the two public supply agency hubs.

2.4. Data processing and analysis

A descriptive frequency and percentage analysis was conducted to evaluate the line-item fill rate, product availability, and distribution trends before and after the COVID-19 pandemic. The following formulas were used to estimate the current product availability, stock out duration, and line-item fill rate of the selected medicines.

For peer review only - http://bmjopen.bmj.com/site/about/guidelines.xhtml

$$\text{Medicine availability} = \frac{\text{Number of product available in a given period}}{\text{Number of products considered.}} \times 100$$

$$\text{Line-item fill rate} = \frac{\text{Amount (quantity) of item received}}{\text{Amount (quantity) of item ordered}} \times 100$$

$$\text{Average stock-out duration} = \frac{\text{Number of days the product was not available}}{\text{Number of products reviewed}} \times 100$$

Based on the WHO's drug availability index, the analysis considered the availability of products that are ordered and received in the supply agencies as well as stock status on the day of data collection. A product is available if it is in stock in the health facility providing the service on the day of the visit or during the specified period. The following ranges were used to describe the medication availability status: less than 30% is very low; 30-49% is low; 50-80% is fairly high; and greater than 80% is very high [25, 26].

2.5. Patient and public involvement

It was not appropriate or possible to involve patients or the public in the design, conduct, reporting, or dissemination plans of our research.

3. Results

3.1. The line-item fill rate

The line-item fill rate indicates how often the EPSA hubs received at least 80% of the quantity ordered for a particular medicine. Prior to COVID-19, the overall average line-item fill rate for the selected products was 78%. It declined to 49% during the pandemic. For antidiabetics, the average line-item fill rate declined from 85% to 56%. For CVD drugs it went down from 76% to 46%. The overall line-fill rate dropped for all selected medicines during COVID-19 (**Figure 1 and 2)**.

3.2. Product availability and stock-out situation of selected basket medicines

During COVID-19, the overall mean (95% CI) of availability of the selected basket medicines was 43.3% (37.1- 49.5). However, it was 67.4 (62.2-72.6) before the outbreak. Comparing the mean availability of the two periods using a two-sample t-test, it showed

For peer review only - http://bmjopen.bmj.com/site/about/guidelines.xhtml

57 a statistically significant difference (P<0.001). On the day of data collection, ten (10) of
58 the twenty-two (46%) were out-of-stock.

59 Concerning the days out of stock, the mean (95% CI) was 11.7 (9.9- 13.5) days before
60 COVID-19 outbreak, which was increased to 15.7 (13.2-18.2) days. This showed a
61 statistically significant difference (P<0.001). Before the pandemic, the average stockout
62 duration per month was 9 days for antidiabetics and 11 days for the CVD drugs. This
63 increased to 13 days and 17 days/month, during the pandemic for antidiabetics and the
64 CVD drugs, respectively. The medicines with the least supply were captopril 25mg
65 tablets, metoprolol 25mg tablets, lovastatin 20mg tablets, and digoxin 0.25mg/ml
66 injection. Lovastatin had the longest average stockout of 28 days, closely followed by
67 digoxin injection at 27 days and captopril tablet at 26 days (resulting in WHO availability
68 indices of 11%, 13 %, and 23%, respectively). The drop in the availability of specific
69 medications ranged from 12% to 77% during the pandemic (**Table 1**).

For peer review only - http://bmjopen.bmj.com/site/about/guidelines.xhtml

Table 1: Product availability and average days out of stock for selected antidiabetics and CVD medications before and during COVID-19 pandemic

List of drug products	Pre-pandemic		During the pandemic		Decline in availability (%)
	Average days out of stock per month	Availability* (%)	Average days out of stock per month	Availability* (%)	
1. Insulin regular (soluble)	9.5	74.7	13.3	44.6	40
2. NPH insulin (suspension)	8	77.3	10.9	49	37
3. Insulin Isophane (biphasic)	8.5	75.2	12.1	43.4	42
4. Metformin 500 mg tablet	8.7	76.5	12.0	47.3	38
5. Glibenclamide 5mg tablet	10.3	70.6	12.3	56.9	19
6. Aspiring 81mg tablet	9.2	70.8	12.7	44	38
7. Enalapril 5mg tablet	11.0	68	14.4	47	31
8. Captopril 25mg tablet	16.7	56	26	23	59
9. Atenolol 50mg tablet	9.6	73.7	11.8	56.4	24
10. Propranolol 40 mg tablet	9.4	72.6	13.2	44	39
11. Metoprolol 25mg tablet	19	52	21.2	46	12
12. Frusemide 40mg tablet	9.3	71.5	12.4	47.7	33
13. Frusemide 10mg/ml	8.4	76	10	61.5	19
14. Hydrochlorothiazide 25mg tablet	10.8	68	16	35	49
15. Spironolactone 25mg tablet	9.6	74	11	63.5	14
16. Atorvastatin 20mg tablet	15	62	20	41.5	33
17. Lovastatin 20mg tablet	17	45	28	10.5	77
18. Methyldopa 250mg tablet	11.3	66.8	17.2	32	52
19. Amlodipine 5mg tablet	8.7	78	11.2	56	28
20. Nifedipine 20mg tablet	13.5	68.3	19.5	38	44
21. Digoxin 0.25mg tablet	9.6	74	12.4	52.5	29
22. Digoxin 0.25mg/ml (injection)	24	31	27	13	58

*Based on the WHO availability index [25, 26].

For peer review only - http://bmjopen.bmj.com/site/about/guidelines.xhtml

3.3. Unit price changes of selected antidiabetics and CVD medications

We were unable to assess the unit pricing changes of all medicines because of stockouts for the majority of the products. The prices were relatively stable for some medicines (where there was sufficient data to assess). Most of these products were from the local pharmaceutical industries. Yet for some of the products, there were dramatic price increases (**Figure 3**), such as for insulin products and methyldopa. Some of these products were imported and supplied by international pharmaceutical suppliers such as Novo Nordisk Pharmatech A/S. Likewise, the unit price for oral furosemide, amlodipine, and nifedipine increased starting from June 2020. During the study period, the value of the Ethiopian birr decreased by 38% compared to United States Dollar (USD) [27], dramatically increasing the price of imported medicines.

3.4. Selected medicine manufacturer/supplier

This paper uses the terms manufacturers and suppliers interchangeably. Sometimes facilities supply their stock from importer and/or wholesaler, sometimes directly from the manufacturer. For the selected NCD medicines, there were nineteen (19) manufacturers/suppliers over the period. During the eight months pre-pandemic, 192 orders were placed by the two agencies while during the 12 months of the pandemic 264 supply orders were placed. Ethiopian companies represented 50% and 53% of suppliers before and during COVID-19 pandemic, respectively. Similarly, European pharmaceutical industries were supplying 27% prior to COVID-19 and 31% during pandemic, meaning that about 84% NCD medicines at the two agencies were supplied either from Ethiopia or Europe during COVID-19 (**Table 2**).

For peer review only - http://bmjopen.bmj.com/site/about/guidelines.xhtml

Table 2 Lists of medicines manufacturer/supplier during & prior to the pandemic.

S. No	Manufacturers/suppliers name (country)	Percentage of supply orders covered	
		Before COVID-19	During COVID-19
1.	Amino AG (Switzerland)	4	8
2.	Addis Pharmaceutical Factory (Ethiopia)	8	13
3.	AstraZeneca (United Kingdom)	4	4
4.	Bliss GVS Pharma Limited (India)	-	3
5.	Brawn Laboratories Limited (India)	4	1
6.	Cadila Pharmaceuticals PLC (Ethiopia)	10	10
7.	Ciron Drugs & Pharmaceuticals PLC (India)	4	4
8.	East African Pharmaceuticals PLC (Ethiopia)	6	10
9.	EIPICO (Egypt)	4	4
10.	Epharm (Ethiopia)	5	1
11.	Gulf Pharmaceuticals Ltd (UAE)	4	4
12.	Humanwell Pharmaceutical PLC (Ethiopia)	4	1
13.	Intas Pharmaceuticals (India)	2	1
14.	Julphar Pharmaceuticals PLC (Ethiopia)	8	5
15.	Laboratoires Sterop (Belgium)	2	1
16.	Novo Nordisk Pharmatech A/S (Denmark)	4	5
17.	Remedica Ltd (Cyprus)	13	13
18.	Reyoung Pharmaceutical Co., Ltd (China)	4	1
19.	Sansheng Pharmaceutical PLC (Ethiopia)	9	13

Epharm: Ethiopian Pharmaceuticals Manufacturing share company EIPICO: Egyptian International Pharmaceutical Industries Company; UAE: United Arab Emirates

For peer review only

For peer review only - http://bmjopen.bmj.com/site/about/guidelines.xhtml

4. Discussion

Ensuring sustainable availability of essential medicines has been a persistent challenge in Ethiopia. The country has made progress in recent years, but the COVID-19 pandemic highlighted the need for further improvements. More must be done to strengthen the drug supply chain and ensure sustainable availability of essential medicines. This study assessed trends in the availability of selected medicines for chronic diseases at the public supply agencies over a long time-period. We evaluated the fill-item rate, trends, and product availability before and during the pandemic.

The results of the study showed that the COVID-19 pandemic significantly increased shortages of NCD medicines at pharmaceutical supply agencies. The researchers found that the stockout situation worsened during the pandemic, indicating challenges in the supply chain of essential medicines. Some of the challenges include disruptions in transportation and logistics, shortages of raw materials and active pharmaceutical ingredients, increased demand for certain medications, and workforce shortages due to illness or quarantine measures. Additionally, border closures and export restrictions have also impacted the availability of essential medicines in some regions. In response to the global burden of NCDs, the WHO has developed a Global Action Plan that includes target of 80% availability and affordability of essential medicines for the prevention and treatment of DM, CVD, and respiratory diseases [28]. Despite this strong recommendation, the availability of NCD medicines in Ethiopia falls below the recommended target. At the national level, the median availability of medicines for NCDs was below 50% with average stockout days of up to 27 days [29]. Similarly, the current study showed that the overall availability of selected medicines was 49% during the pandemic, down from 78% pre-pandemic. This includes wide variation, with lovastatin available only 11% according to the WHO availability index during the pandemic. This is consistent with studies from Kenya [30], Portugal [31] , Poland [32] and Congo and Cameroon [33]. This has led to increased difficulties for individuals with chronic conditions to access the medications they need to manage their health. The higher rates of stockouts mean that pharmacies and healthcare facilities are unable to meet the demand for these essential medicines, leaving patients without access to necessary treatments.

For peer review only - http://bmjopen.bmj.com/site/about/guidelines.xhtml

Not only were the medicines less available during COVID-19 but some also became more expensive, which is in line with several studies from countries like Bangladesh [34], Portugal [31], Saudi Arabia [35], India [36], Nigeria [37] and Namibia [38]. While the Ethiopian Birr decreased in value compared to the US Dollar by 38%, soluble insulin increased by more than 130% in price, which means exchange rates cannot alone explain the price increase. This suggests that other factors, such as supply chain disruptions or increased production costs, may have contributed to the price hike. Moreover, the changes in supplier dynamics on medication availability and affordability might contribute to the price changes during the pandemic. These findings highlight the need for further investigation into the underlying causes of medication price increases during crises. It is crucial to understand the specific factors influencing these price changes to develop effective strategies for mitigating their impact on individuals with chronic conditions. This study also suggests that while there were stable prices for certain medicines supplied by local pharmaceutical industries, there were price increases for insulin products and methyldopa, which were imported and supplied by international pharmaceutical suppliers. Factors such as supply chain disruptions, increased demand, and changes in production costs could contribute to these price fluctuations. It is important to further investigate the reasons behind these price increases, as they may have implications for medication affordability and accessibility.

Furthermore, this study also assessed the type of suppliers or manufacturers of selected drugs before and during the epidemic. The supply distribution did not vary substantially by region during and after COVID-19. For NCD medicines it appears that, at least these two public pharmaceutical supply agencies, are mainly supplied by Ethiopian and European companies. The large proportion of Ethiopian suppliers is beneficial, likely both in normal times and in pandemic, as the currency fluctuations should be smaller, although still present due to a likely dependence on imported active pharmaceutical ingredients (APIs). That local pharmaceutical industries were able to step up and meet the demand for these products demonstrates the importance of having strong and resilient local pharmaceutical industries that can provide essential healthcare products during times of crisis. This study highlights the importance of addressing supply chain disruptions during public health emergencies, such as the COVID-19 pandemic, to ensure the availability of

For peer review only - http://bmjopen.bmj.com/site/about/guidelines.xhtml

essential medicines for patients with NCDs. It emphasizes the need for interventions and strategies to strengthen the pharmaceutical supply chain and improve access to essential medicines in low-income countries. Overall, the COVID-19 pandemic has underscored the critical importance of maintaining a robust and resilient healthcare system that can effectively respond to emergencies while ensuring the availability and affordability of essential medicines for those who need them most.

Limitation of the study

To the best of the authors' knowledge, this is one of the first studies assessing supply situations of essential medicines during a pandemic at selected public pharmaceutical supply agencies. Yet, the results may not apply to all medicines. Other therapeutic areas may experience unique trends. The study included only two facilities, which may have unique characteristics.

5. Conclusion

In summary, significant shortages of selected basket medicines used for the management of DM and CVD were observed at the two selected public pharmaceutical supply agencies. The pandemic has highlighted the importance of ensuring access to essential medications for patients with chronic conditions such as CVD and diabetes. Unit prices of some of the medicines dramatically increased, due to both currency fluctuations and other possible factors. The supply distribution did not vary substantially by region during and after COVID-19, with the two agencies mainly being supplied by Ethiopian and European companies. The findings of this study suggest that the COVID-19 pandemic has exacerbated existing medicines availability problems. Yet it also demonstrates a strong local provision of NCD medicines. Challenges facing these local providers should be further studied to understand how currency fluctuations, dependencies on imported APIs, and other factors influence their ability to supply the local population. It would also be useful to compare these findings with patient experiences to better understand how these stockouts impacted them and their health.

For peer review only - http://bmjopen.bmj.com/site/about/guidelines.xhtml

Abbreviations: APIs: Active Pharmaceutical Ingredients; CVD: Cardiovascular Diseases; COVID-19: coronavirus disease 2019; EPSA: Ethiopian Pharmaceutical Supply Agency; IRB: Institutional Review Board; SPSS: Statistical Package Software for Social Science; LMICs: low- and middle-income countries; NCDs: Non-communicable Diseases; IPLS: Integrated Pharmaceutical and Logistic Services; USD: United States Dollar; WHO: World Health Organization

Declarations

Ethics approval and consent to participate: Before commencing data collection, ethical approval was obtained from the Institutional Review Board (IRB) of the Institute of Health, Jimma University in Ethiopia (reference number JHRPG/1043/2020). Then, the selected public supply agencies were communicated with a formal letter from the health research and innovation office of Jimma University. The study was conducted after getting permission from the manager of EPSA.

Consent for publication: Not required.

Availability of data and materials: This study included all pertinent data. Additional information could be obtained from the corresponding author upon reasonable request.

Competing interests: None declared.

Funding: This work was supported by the Global Health and vaccination research programme

(GLOBVAC), which is the programme of the Research Council of Norway.

Authors' contribution: ZM, TSM, GTT, MM, CÅ, and MJ designed the study. ZM and TSM drafted the first version of the manuscript. ZM, TSM, CÅ, and MJ discussed the analytic strategy. ZM, TSM, and GTT did the data curation and performed the analysis. All authors reviewed the final paper and made creative and substantial contributions, approved the final version, and consented to publication.

Acknowledgements: We would like to extend our appreciation and thank our data collectors and study participants. We also extend our sincere gratitude to health care professionals, and managers at supply agency hubs for providing us with necessary

For peer review only - http://bmjopen.bmj.com/site/about/guidelines.xhtml

data/information, and for their kind cooperation, and assistance throughout the data collection process. Additionally, we would like to express our heartfelt gratitude to the Global Health and Vaccination Research Programme (GLOBVAC) for financially supporting this research under the auspices of the MIA (Measures for Improved Availability of Medicines and Vaccines (The MIA Project | BI). Finally, our acknowledgment extends to the Institute of Health of Jimma University for supporting us in the ethical approval process and in the official communication of respective offices and health facilities.

References

1. Hogerzeil, H.V., *Essential medicines and human rights: what can they learn from each other?* Bulletin of the World Health Organization, 2006. **84**: p. 371-375.
2. De Weerdt, E., et al., *Toward a European definition for a drug shortage: a qualitative study.* Frontiers in pharmacology, 2015. **6**: p. 253.
3. Wagenaar, B.H., et al., *Stock-outs of essential health products in M ozambique–longitudinal analyses from 2011 to 2013.* Tropical Medicine & International Health, 2014. **19**(7): p. 791-801.
4. Wild, L. and D. Cammack, *The supply and distribution of essential medicines in Malawi 2013.* https://www.refworld.org/pdfid/523acf304.pdf; *access date: 13* March 2023.
5. Kohler, J.C., et al., *Corruption in the pharmaceutical sector: diagnosing the challenges.* https://www.transparency.org.uk/sites/default/files/pdf/publications/29-06-2016-Corruption_In_The_Pharmaceutical_Sector_Web-2.pdf ; *access date: 13* March 2023.
6. Martinez, M.G., J.C. Kohler, and H. McAlister, *Corruption in the pharmaceutical sector*, in *The Handbook of Business and Corruption*. 2017, Emerald Publishing Limited. p. 329-361.
7. Besancon, L. and B. Chaar, *Report of the International Summit on medicines shortage.* Toronto, Canada: International Pharmaceutical Federation; https://www.fip.org/files/fip/publications/FIP_Summit_on_Medicines_Shortage.pdf; *access date: 18* April 2023.
8. Morrison, A., *Drug supply disruptions [environmental scan issue 17].* Ottawa: Canadian Agency for Drugs and Technologies in Health. Available online at: https://www.cadth.ca/drug-supply-disruptions; *access date: 18* April 2023.
9. World Health Organization, *Access to medicines: making market forces serve the poor*; https://www.who.int/publications/m/item/access-to-medicines-making-market-forces-serve-the-poor; *access date: 20* April 2023.
10. Ahlqvist, V., et al., *Supply chain risk management strategies in normal and abnormal times: policymakers' role in reducing generic medicine shortages.* International Journal of Physical Distribution & Logistics Management, 2023. **53**(2): p. 206-230.
11. Magableh, G.M., *Supply chains and the COVID-19 pandemic: A comprehensive framework.* European Management Review, 2021. **18**(3): p. 363-382.
12. Yu, Z., et al., *Disruption in global supply chain and socio-economic shocks: a lesson from COVID-19 for sustainable production and consumption.* Operations Management Research, 2021: p. 1-16.
13. Ziavrou, K.S., S. Noguera, and V.A. Boumba, *Trends in counterfeit drugs and pharmaceuticals before and during COVID-19 pandemic.* Forensic Science International, 2022: p. 111382.

For peer review only - http://bmjopen.bmj.com/site/about/guidelines.xhtml

14. Noguera, S. and K. Ziavrou, *With the world in turmoil, transnational organized crime sees opportunity in counterfeit pharmaceuticals and medical supplies during the COVID-19 pandemic.* Perspectives, 2020. **12**.
15. Ozili, P.K. and T. Arun, *Spillover of COVID-19: impact on the Global Economy.* Available at SSRN 3562570, 2020.
16. Bookwalter, C.M., *Drug shortages amid the COVID-19 pandemic.* Us Pharm, 2021. **46**(2): p. 25-8.
17. World Health Organization, *COVID-19 pandemic significantly impacted access to medicines for noncommunicable diseases;* https://www.who.int/news/item/22-03-2023-covid-19-pandemic-significantly-impacted-access-to-medicines-for-noncommunicable-diseases *; access date 20 April 2023.*
18. World Health Organization, *Access to NCD medicines: emergent issues during the COVID-19 pandemic and key structural factors;* https://reliefweb.int/report/world/access-ncd-medicines-emergent-issues-during-covid-19-pandemic-and-key-structural-factors*; access date 25 April 2023.*
19. Sen-Crowe, B., M. McKenney, and A. Elkbuli, *Medication shortages during the COVID-19 pandemic: Saving more than COVID lives.* The American journal of emergency medicine, 2021. **45**: p. 557.
20. Mekonnen, Z., et al., *The knock-on effects of COVID-19 pandemic on the supply and availability of generic medicines in Ethiopia: mixed methods study.* BMC Health Services Research, 2023. **23**(1): p. 1-16.
21. Agency, E.P.S., *Info Hubs - Ethiopian Pharmaceuticals Supply Agency(EPSA);* https://epss.gov.et/info-hubs/ *; Accessed date: May, 22, 2023*
22. Management Science for Health (2012); Chapter 22-Managing distribution; available from https://msh.org/wp-content/uploads/2013/04/mds3-ch22-distribution-mar2012.pdf: accessed date 4 December 2023.
23. United States Agency for International Development (USAID)/DELIVER PROJECT; *Logistics Indicators Assessment Tool (LIAT).* 2008, https://pdf.usaid.gov/pdf_docs/Pnade735.pdf; access date 25 September 2021
24. United States Agency for International Development (USAID)/DELIVER PROJECT; *Logistics System Assessment Tool (LSAT).* 2009; https://pdf.usaid.gov/pdf_docs/Pnado527.pdf; access date 25 September 2021
25. World Health Organization, *Measuring medicine prices, availability, affordability and price components*. 2008, https://www.who.int/publications-detail-redirect/WHO-PSM-PAR-2008.3 ; *accessed date: 20 May 2023.*
26. World Health Organization, *Technical definitions of shortages and Stockouts of medicines and vaccines.* Geneva: World Health Organization, 2017. https://cdn.who.int/media/docs/default-source/medicines/meeting_report_october_shortages.pdf?sfvrsn=1a902eab_3&download=true ; *accessed date: 20 May 2023.*
27. Exchange-Rates.org, *US Dollar (USD) To Ethiopian Birr (ETB) Exchange Rate History for December 31, 2019;* *https://www.exchange-rates.org/exchange-rate-history/usd-etb-2019-12-31*. *accessed date: 20 May 2023.*
28. World Health Organization, *Global action plan for the prevention and control of noncommunicable diseases 2013-2020*; https://www.who.int/publications-detail-redirect/9789241506236 *; access date 25 April 2023.*
29. World Health Organization, Ethiopian Food and drug Authority; *Assessment of the pharmaceutical sector in Ethiopia*. 2017 http://www.fmhaca.gov.et/wp-content/uploads/2019/03/Pharmaceutical-Assessment-2016.pdf . accessed date: 23 May 2023.
30. Kiragu, Z.W., et al., *Household access to non-communicable disease medicines during universal health care roll-out in Kenya: A time series analysis.* PloS one, 2022. **17**(4): p. e0266715.

For peer review only

For peer review only - http://bmjopen.bmj.com/site/about/guidelines.xhtml

31. Romano, S., et al., *Time-trend analysis of medicine sales and shortages during COVID-19 outbreak: Data from community pharmacies.* Research in Social and Administrative Pharmacy, 2021. **17**(1): p. 1876-1881.
32. Nowak, B.M., et al., *Availability of Cardiodiabetological Drugs in Poland during the First Year of COVID-19 Pandemic: Retrospective Study.* BioMed, 2022. **2**(1): p. 117-126.
33. Schäfermann, S., et al., *Availability, prices and affordability of selected antibiotics and medicines against non-communicable diseases in western Cameroon and northeast DR Congo.* Plos one, 2020. **15**(1): p. e0227515.
34. Haque, M., et al., *Availability and price changes of potential medicines and equipment for the prevention and treatment of COVID-19 among pharmacy and drug stores in Bangladesh; findings and implications.* Bangladesh Journal of Medical Science, 2020: p. 36-S 50.
35. Aljadeed, R., et al. *The impact of COVID-19 on essential medicines and personal protective equipment availability and prices in Saudi Arabia*. in *Healthcare*. 2021. MDPI.
36. Haque, M., et al., *Utilisation, availability and price changes of medicines and protection equipment for COVID-19 among selected regions in India: findings and implications.* Frontiers in pharmacology, 2021. **11**: p. 582154.
37. Awucha, N.E., et al., *Impact of the COVID-19 pandemic on consumers' access to essential medicines in Nigeria.* The American journal of tropical medicine and hygiene, 2020. **103**(4): p. 1630.
38. Tirivangani, T., et al., Impact of COVID-19 pandemic on pharmaceutical systems and supply chain–a phenomenological study. Exploratory Research in clinical and social pharmacy, 2021. **2:** p. 100037.

Figure 1: The average line-item fill rate for selected antidiabetics before and during COVID-19

Figure 2: The average line-item fill rate for selected CVD medications before and during COVID-19.

Figure 3: Trends of selected antidiabetics (A, B) and CVD medicines (C, D) unit prices at public pharmaceutical supply agency hub

For peer review only - http://bmjopen.bmj.com/site/about/guidelines.xhtml

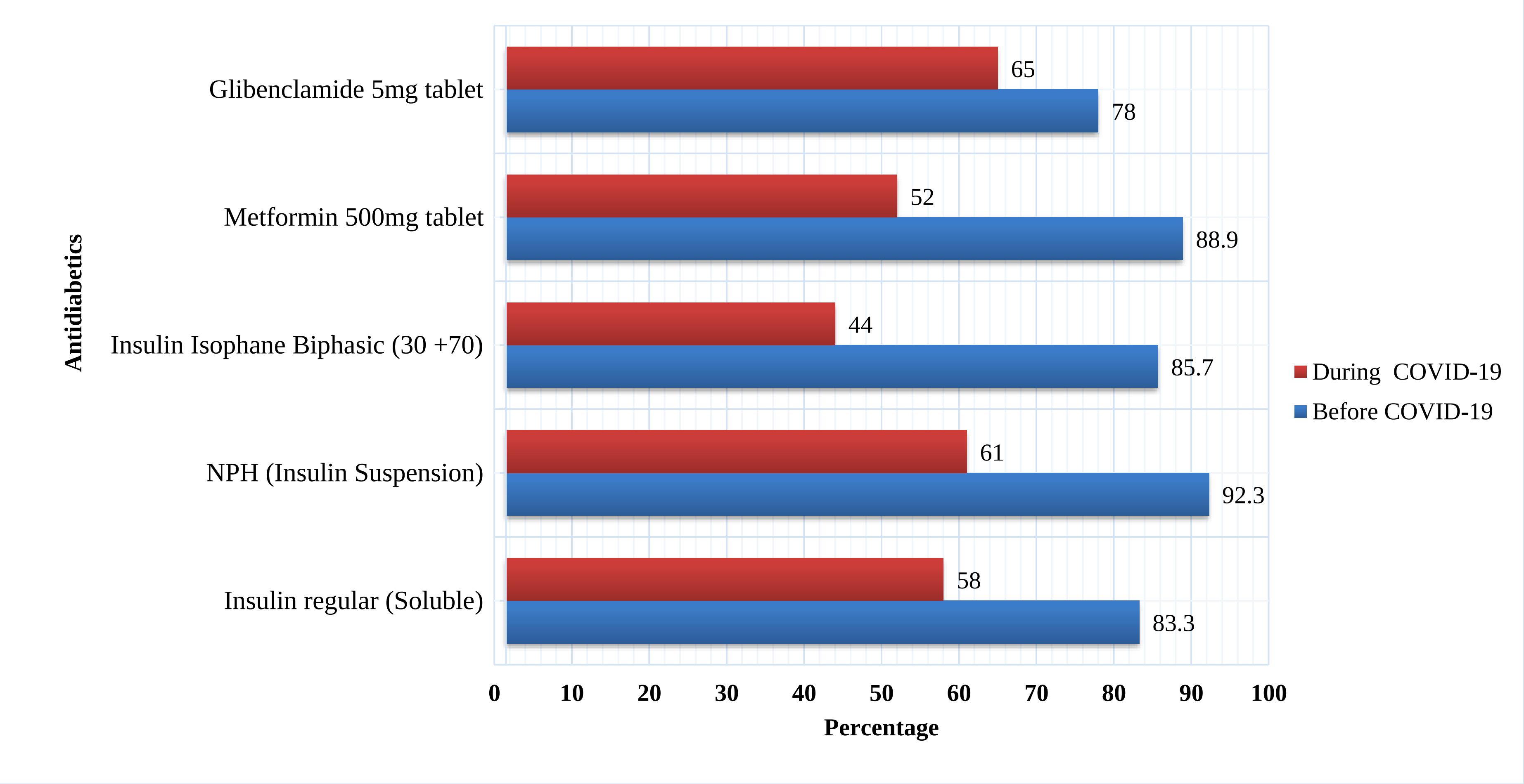

Figure 1: The average line-item fill rate for selected antidiabetics before and during COVID-19

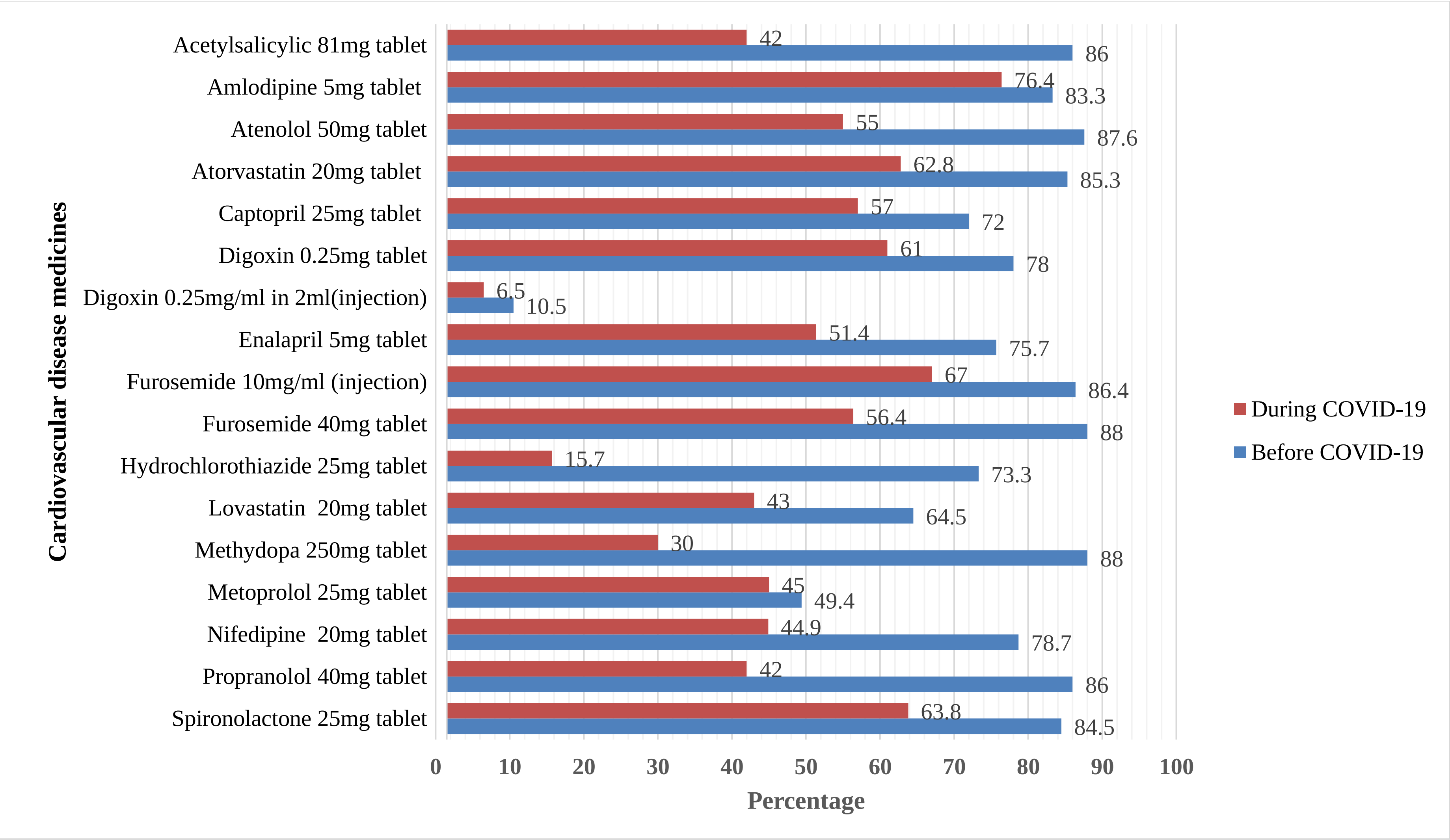

Figure 2: The average line-item fill rate for selected CVD medications before and during COVID-19.

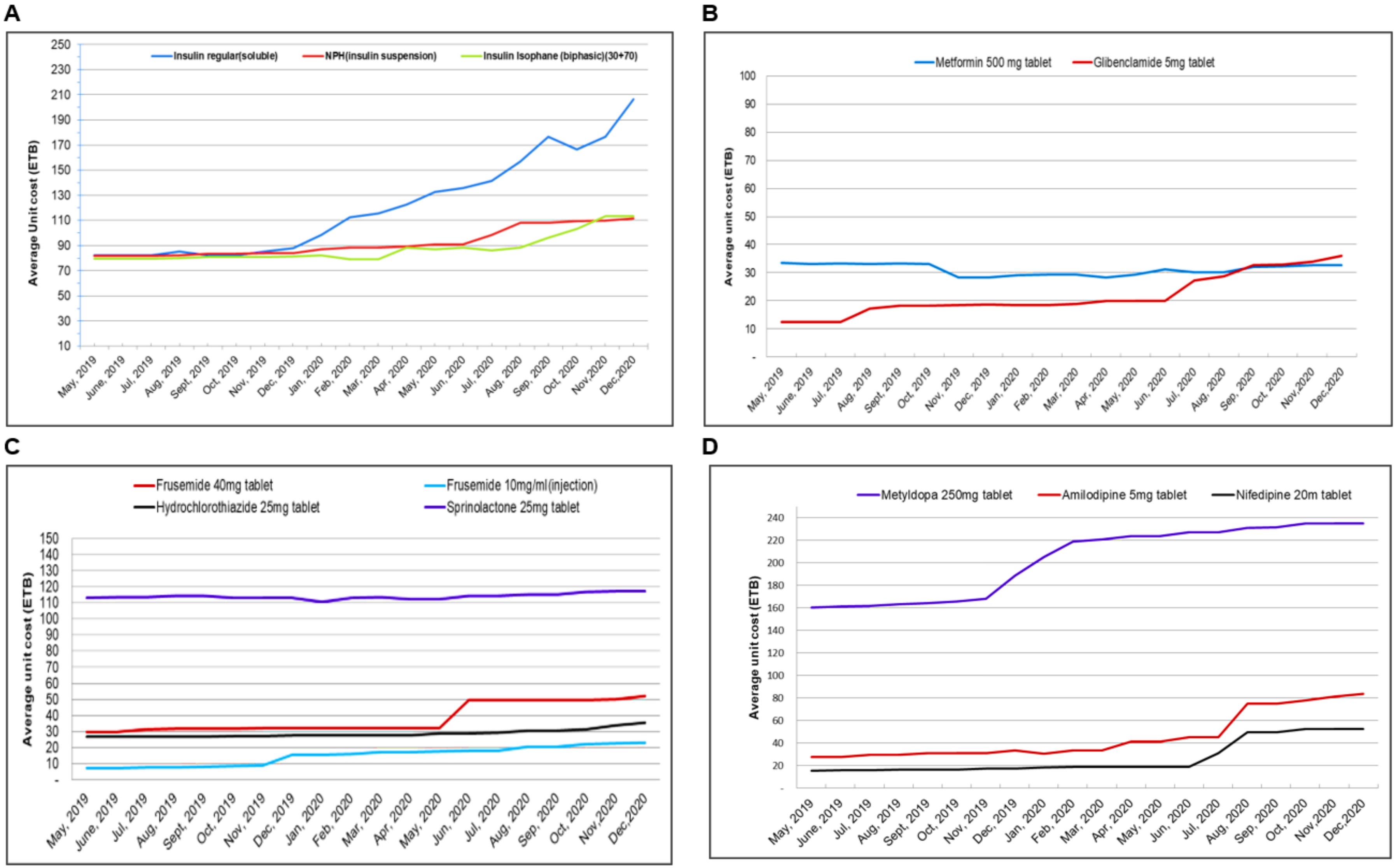

Figure 3: Trends of selected antidiabetics (A, B) and CVD medicines (C, D) unit prices at public pharmaceutical supply agency hub
